# Fast hydrated-ion transport and desolvation in pyridinyl COF membranes *via* competitive coordination

**DOI:** 10.1039/d6sc01290f

**Published:** 2026-04-13

**Authors:** Xuan Li, Jiaxin Liu, Yilin Zhang, Zhixiang Dai, Zihan Tan, Hongli Yang, Chao Xu, Maria Strømme, Shengyang Zhou, Zhong-Ming Li

**Affiliations:** a College of Materials Science and Engineering, College of Polymer Science and Engineering, State Key Laboratory of Advanced Polymer Materials, Sichuan University Chengdu 610065 China shengyang.zhou@scu.edu.cn; b Department of Materials Science and Engineering, The Ångström Laboratory, Uppsala University Uppsala 752 37 Sweden

## Abstract

Hydrated-ion transport and desolvation dominate energy transfer and ionic selectivity in membrane separation, electrochemical energy storage, and catalytic systems, whereas achieving fast ion conduction with low hydration remains highly challenging. In this work, we discover that pyridinyl-based covalent organic framework (COF) membranes enable the fast transport of hydrated ions with efficient desolvation. This originates from a soft Lewis acid-mediated competitive coordination mechanism, where pyridinyl groups partially displace hydration shells. The ordered channels with optimized coordination not only stabilize desolvated ions but also provide continuous hopping pathways for ion migration, resulting in rapid ion transport with reduced desolvation barriers. As a proof-of-concept application, the pyridinyl COF membranes were studied in aqueous zinc batteries. Electrochemical tests reveal that partial zinc ion desolvation lowers the thermodynamic barrier for zinc nucleation, while rapid ion transport balances interfacial reaction kinetics, effectively suppressing dendrite growth and parasitic reactions. Consequently, a zinc anode paired with pyridyl COF membranes exhibits reversible stripping/plating over 2000 cycles in a conventional ZnSO_4_ electrolyte without additives, outperforming most of the current aqueous battery separators. This work demonstrates the unique desolvation transport behavior of hydrated ions in pyridinyl COF membranes and provides new insights for the rational design of COFs for electrochemical energy storage.

## Abstract 2

This work demonstrates that pyridinyl COF membranes enable partial desolvation and rapid hydrated-ion transport *via* competitive coordination, thereby enhancing the cycling stability of zinc-ion batteries.

## Graphical abstract

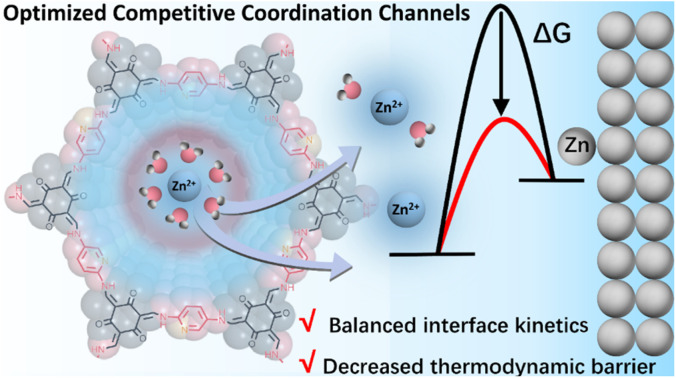

## Introduction

The regulation of hydrated ions is fundamental to a wide range of energy storage,^1,2^ chemical separation engineering,^3,4^ and catalytic systems^5,6^ since ion transport and desolvation directly control energy transfer, ionic selectivity, and reaction kinetics.^7,8^ In membrane systems, precise control of ion coordination environments and migration pathways determines the efficiency and specificity of ion transport and its overall performance.^9–11^ This modulation becomes particularly critical in electrochemical energy storage devices such as aqueous batteries, where hydrated ions must traverse both the electrolyte and electrode interfaces, and their solvation structures directly influence the thermodynamics and kinetics of electrochemical reactions.^12,13^ For example, Zn^2+^ in aqueous zinc-ion batteries exists in fully hydrated states, and the energy required to partially remove water molecules from their hydration shell directly affects nucleation overpotential, deposition uniformity, and the likelihood of dendrite formation.^14–17^ Rapid ion conduction under low hydration conditions is therefore essential to maintain uniform ion flux, enhance charge transfer, and suppress parasitic reactions at the electrode–electrolyte interface. Achieving both low solvation and high mobility is particularly challenging because de-hydration processes are energetically demanding. Conventional membranes rarely provide the chemical functionality and well-defined pore topology necessary to facilitate ion desolvation and stabilize partially desolvated ions. They also lack the ordered architecture required to guide the directional transport of partially desolvated ions and to reduce the thermodynamic barriers associated with their low-solvation state and limited stability.^18,19^ Consequently, the rational design of materials capable of efficient low-hydrated ion transport remains a major scientific and engineering challenge with broad implications for improving the efficiency, stability, and safety of electrochemical energy storage devices and for advancing catalysis, selective ion separation, and other processes dependent on controlled ion transport.

**Scheme 1:**
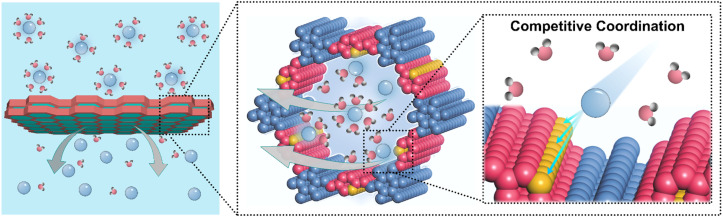
Schematic illustration of pyridinyl COF membranes for rapid transport and partial desolvation of hydrated ions *via* a competitive coordination mechanism.

In this work, we find that pyridinyl-functionalized covalent organic framework (COF) membranes can realize the rapid transmembrane transport of hydrated ions while facilitating their partial desolvation. This behavior arises from a soft Lewis acid-mediated competitive coordination mechanism in which pyridinyl groups selectively interact with hydrated ions and partially displace water molecules from their hydration shells (Scheme 1). Such regulated desolvation is cooperatively supported by the spatial confinement and structural ordering of the COF nanochannels, which stabilize partially desolvated ions and reduce the energetic barrier associated with low solvation transport, thereby enabling directional and efficient ion conduction. To elucidate the roles of pyridinyl coordination and pore topology in this process, two structurally related COF membranes containing single pyridine and bipyridine units were designed and fabricated. Comparative analysis reveals that the single pyridine COF membrane establishes a moderate competitive coordination environment within confined nanochannels. This environment enables effective ion desolvation, accelerates diffusion, and preserves thermodynamic stability. In contrast, the bipyridine COF exhibits excessive coordination that leads to ion immobilization and slowed migration, thereby lowering desolvation efficiency. As a conceptual validation of this transmembrane ion regulation strategy, both COF membranes were evaluated in aqueous zinc battery (AZB) systems. Electrochemical results demonstrate that regulated zinc ion desolvation and transport within confined nanochannels lower the thermodynamic barrier for Zn nucleation and balance the kinetic rates of ion diffusion and surface deposition. This synergy ensures uniform ion flux, promotes homogeneous zinc growth, and effectively suppresses dendrite formation and parasitic reactions such as hydrogen evolution and local hydroxide accumulation. Consequently, zinc anodes paired with the single pyridine COF membrane exhibit stable stripping and plating over 2000 cycles (>5000 hours) in a conventional 2 M ZnSO_4_ electrolyte without additives, obviously outperforming most currently used separators in AZBs. These results show that the rational design of chemical sites combined with the highly ordered nanochannel architecture of COF membranes provides an effective platform to couple ion solvation thermodynamics with transport kinetics. They also highlight the potential of COFs for the development of next-generation ion-regulating membranes and offer theoretical inspiration for their broader applications in electrochemical energy storage, separation technology, catalysis engineering, and membrane-based ion regulation.

## Results and discussion

**Fig. 1:**
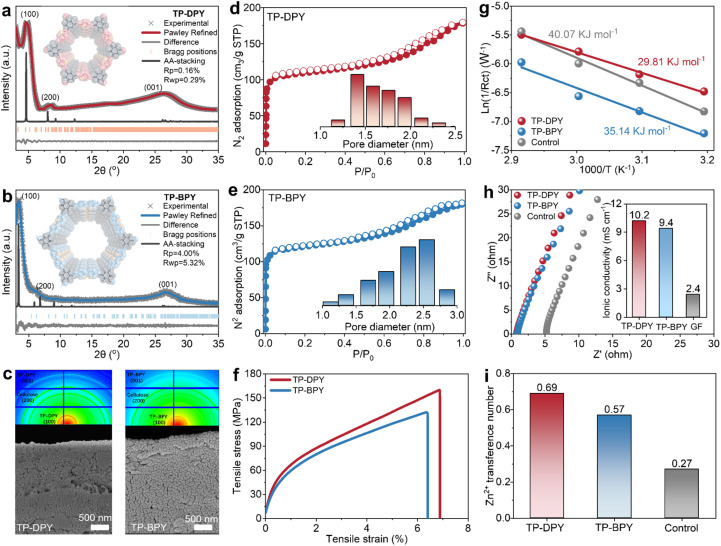
Structural characterization and hydrated ion transport properties of two pyridine COF membranes. Experimental and simulated XRD patterns with corresponding molecular models of (a) TP-DPY and (b) TP-BPY COFs. (c and d) N_2_ sorption isotherms and calculated pore size distributions, (e) SEM images and corresponding 2D-WAXS patterns, and (f) mechanical tensile stress–strain curves of the two COF membranes. (g) Arrhenius activation energy, (h) Nyquist plots and calculated ionic conductivity, and (i) zinc ion transference numbers of the two COF membranes and bulk ZnSO_4_ electrolyte as the control, respectively.

Two pyridine-based COFs, TP-DPY and TP-BPY, were synthesized *via* a one-step condensation reaction of 2,4,6-triformylphloroglucinol (TP) with 2,6-diaminopyridine (DPY) or 2,2′-bipyridine-5,5′-diamine (BPY), as illustrated in Fig. S1 and S2.^20,21^ An aqueous synthesis route was employed using water as the solvent and acetic acid as the catalyst, representing an environmentally friendly approach to conventional solvothermal methods that rely on organic solvents. This method allowed the formation of highly crystalline COFs under ambient temperature and pressure conditions, as confirmed by X-ray diffraction patterns in Fig. 1a and b. Both COFs are constructed through β-ketoenamine linkages, which impart robust chemical stability and hydrolytic resistance, making them suitable for prolonged operation in aqueous environments.^20,21^ The formation of the two pyridyl-based COFs was further confirmed by multiple spectroscopic techniques. Solid-state ^13^C nuclear magnetic resonance (^13^C NMR) and Fourier-transform infrared (FTIR) spectroscopy show the characteristic signals of C=O and C–N, consistent with the typical chemical structure of β-ketoenamine linked COFs (Fig. S3 and S4). For membrane fabrication, nanocellulose was incorporated as a binder, and a vacuum-assisted filtration technique was applied (Fig. S5).^22^ This straightforward procedure yielded freestanding and flexible membranes from COF powders with uniform thickness (Fig. S6). Scanning electron microscopy (SEM) images reveal dense and homogeneously packed COF particles within both membranes, while two-dimensional wide-angle X-ray scattering (2D-WAXS) and energy dispersive spectroscopy (EDS) analyses confirm an isotropic distribution of COF crystals, highlighting the uniformity of the membrane microstructure (Fig. 1c and S6–S8). Nitrogen adsorption analysis indicates a highly porous structure of these two COF membranes dominated by micropores of 1–2 nm, arising from the intrinsic nanopores of the COF skeleton (Fig. 1d and e). Mechanical characterization demonstrated that both COF membranes exhibit tensile strengths of over 100 MPa (Fig. 1f), which are substantially higher than those of COF membranes prepared *via* conventional two-phase interfacial methods that typically show tensile strengths below 5 MPa. The combination of highly ordered, uniform nanochannels and robust mechanical integrity renders these two COF membranes an ideal and stable platform for electrochemical investigations, providing well-defined confined pathways for ion transport under practical operating conditions.

Water contact angle measurements reveal that both pyridine-based COF membranes exhibit initial contact angles of approximately 40–50°. After ten minutes of contact, the angles decrease to below 20°, confirming their obvious hydrophilic nature (Fig. S9). This wetting behavior is essential for ensuring uniform and stable electrolyte infiltration for reliable operation in aqueous environments. The zinc ion transport properties of the two COF membranes were systematically evaluated in comparison with the bulk 2 M ZnSO_4_ electrolyte as a control. As shown in Fig. 1g, the Arrhenius activation energy is substantially lower for the TP-DPY and TP-BPY membranes than for the bulk electrolyte, decreasing from 40.07 kJ mol^−1^ to 29.81 kJ mol^−1^ and 35.14 kJ mol^−1^, respectively (Fig. S10 and S11 and Table S2). This decrease in activation energy reflects accelerated Zn^2+^ migration within the ordered COF channels and indicates improved ion transport kinetics compared with the bulk electrolyte. As a result, both COF membranes exhibit zinc ion conductivities approaching up to ∼10 mS cm^−1^, which is four times higher than that of the bulk electrolyte with 2.4 mS cm^−1^ (Fig. 1h). COF membrane thickness has little effect on the Zn^2+^ ionic conductivity of the COF membranes (Fig. S12). In addition, the zinc ion transference numbers also show remarkable improvement, reaching 0.69 and 0.57 for both TP-DPY and TP-BPY membranes, respectively, compared with 0.27 for the bulk electrolyte (Fig. 1i and S13). The reduced Arrhenius activation energy, together with the enhanced Zn^2+^ conductivity and transference number, demonstrates that the COF membranes establish cation-selective, low-barrier transport pathways, thereby accelerating Zn^2+^ transport kinetics. This behavior confirms that Zn^2+^ serves as the dominant charge carrier, with more efficient and selective ion conduction within the COF membranes.

**Fig. 2:**
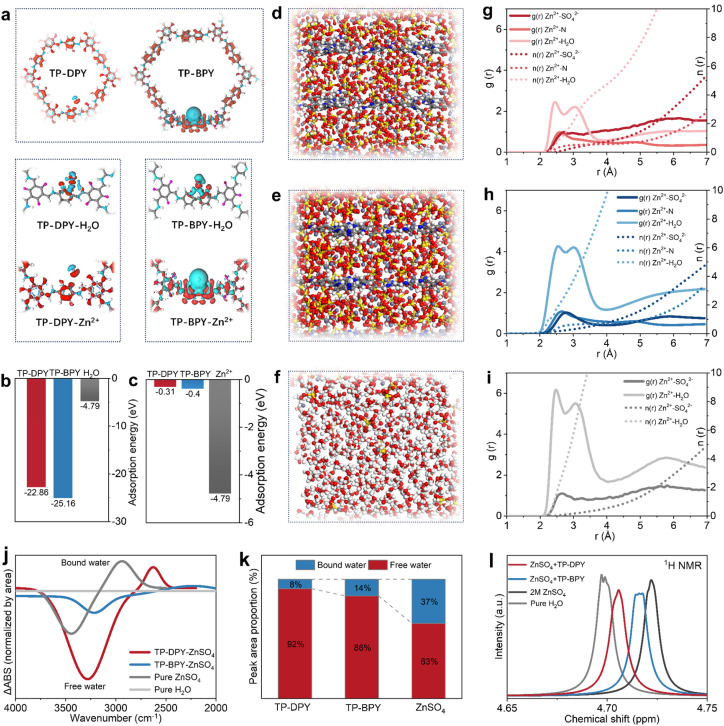
Mechanistic insights into hydrated ion transport behaviors in pyridinyl COF membranes. (a) Charge density difference profiles and corresponding enlarged views of TP-DPY and TP-BPY calculated using density functional theory (DFT). Calculated interaction energies of (b) Zn^2+^ and (c) H_2_O with different molecular structures. Molecular dynamics (MD) simulation snapshots of ZnSO_4_ electrolyte within (d) TP-DPY, (e) TP-BPY, and (f) bulk solution. Simulated radial distribution functions (RDFs) and coordination numbers (CNs) of Zn^2+^ in (g) TP-DPY, (h) TP-BPY, and (i) bulk ZnSO_4_ electrolyte. (j) FTIR and (l) ^1^H NMR spectra of pure water and 2 M ZnSO_4_ electrolytes within different membranes. (k) The corresponding proportions of hydrogen bonds calculated based on FTIR spectra.

To investigate the origin of the low migration energy barrier and the unusual transport behavior of hydrated ions within the COF membranes, density functional theory (DFT) calculations were conducted to systematically decouple and quantify the interactions of the two pyridinyl COFs with zinc ions and water molecules.^23–26^ We first calculated the charge density difference by subtracting the sum of the charge densities of the isolated COFs and Zn^2+^ from that of the combined COFs-Zn^2+^ system, thereby revealing the redistribution of electrons during their interaction. The results show that bipyridyl exhibits stronger electron donation and more pronounced charge polarization compared to mono-pyridyl, as shown in Fig. 2a. The calculated adsorption energies suggest that pyridyl COFs bind Zn^2+^ significantly more strongly than Zn^2+^ interacts with water in bulk electrolyte (Fig. 2b and c). This indicates that strong interactions with pyridinyl groups can thermodynamically remodel the local coordination environment of hydrated Zn^2+^ ions during their transport through the COF channels. Meanwhile, we found that the adsorption energy of Zn^2+^ on the mono-pyridyl COFs (22.86 eV) is lower than that on the bipyridyl COFs (25.16 eV), indicating that binding strength is not positively correlated with ion conduction capability. This behavior actually aligns with the hopping mechanism in ion conduction, in which the moderate Zn^2+^ coordination in mono-pyridyl COFs promotes dynamic hopping by enabling fast coordination exchange along the channels, in agreement with the experimentally observed reduction in Arrhenius activation energy and increase in the Zn^2+^ transference number in mono-pyridyl COF membranes.

Based on the above results, we further conducted molecular dynamics (MD) simulations to investigate the fine coordination environment and dynamic interactions of hydrated Zn^2+^ ions confined within the nanochannels of TP-DPY and TP-BPY COF membranes.^27–29^ To better model the state of hydrated ions within COF membranes, multilayer COF crystal models were constructed. A 2 M ZnSO_4_ aqueous solution was then introduced into the nanopores of the two pyridinyl COF membranes (Fig. 2d–f and S14–16). Periodic boundary conditions were applied in all three dimensions, and simulations were performed at 298 K and 1 atm to allow equilibration of the solvation structures. The force fields for the COFs and ions were refined *via* DFT-based charge fitting to accurately capture interactions between Zn^2+^ and the chemical groups. Simulation results reveal that zinc ions migrate significantly faster in TP-DPY COF membranes compared to TP-BPY COF membranes. Meanwhile, the average coordination number of Zn^2+^ in TP-DPY COF membranes is obviously lower than that in TP-BPY COF membranes and bulk electrolyte (Fig. 2g–i), reflecting a more labile and dynamically fluctuating hydration environment. This can be explained within the framework of hopping-based ion conduction because the moderate binding strength of mono-pyridyl groups facilitates rapid coordination and de-coordination events, which allow hydrated zinc ions to efficiently hop between pyridinyl sites. The high mobility naturally leads to a lower statistical average coordination number, as zinc ions do not remain fully coordinated at any single site for extended periods. In contrast, stronger bipyridyl interactions in TP-BPY COFs kinetically trap zinc ions, rigidify the local solvation structure, reduce hopping frequency, and slow overall ion transport.

The desolvation behavior in pyridine-based COF membranes was further experimentally confirmed by probing the state of water molecules using FTIR spectroscopy (Fig. 2j, k, S17 and S18) and NMR spectroscopy (Fig. 2l). Using ultrapure water as the reference, infrared difference spectroscopy was applied to the O–H stretching region from 4000 to 2000 cm^−1^ to quantitatively compare the relative fractions of free and bound water in bulk ZnSO_4_ electrolyte and within two pyridinyl COF membranes, as shown in Fig. 2j, with the calculated results summarized in Fig. 2k. In bulk ZnSO_4_ electrolyte, approximately 37% of water exists in a coordinated bound state, whereas the fraction of bound water decreases markedly to 14% in TP-BPY and further to 8% in TP-DPY. This reduction indicates that confinement within pyridinyl COF channels effectively weakens Zn^2+^–water coordination and promotes partial disruption of the hydration structure. To further probe the state of water molecules in different environments, the ^1^H NMR spectrum was obtained, as shown in Fig. 2l. In bulk electrolyte, coordination of water to Zn^2+^ leads to a pronounced downfield shift to higher ppm values due to proton de-shielding induced by strong electrostatic interactions with the cation. In contrast, when hydrated Zn^2+^ ions are confined within pyridinyl COF membranes, the water proton signals shift up-field toward lower ppm values that are closer to those of pure water, reflecting weakened Zn^2+^–water interactions and a more labile solvation environment arising from competitive coordination with pyridinyl groups. These spectroscopic results provide direct experimental evidence that pyridinyl COF membranes can induce partial desolvation of Zn^2+^ ions, thereby decreasing the energetic barrier associated with ion transport. The more pronounced reduction of bound water in TP-DPY further highlights the advantage of mono-pyridyl units within membranes, whose moderate coordination strength facilitates efficient Zn^2+^ migration by balancing desolvation and dynamic hopping within the COF channels.

**Fig. 3:**
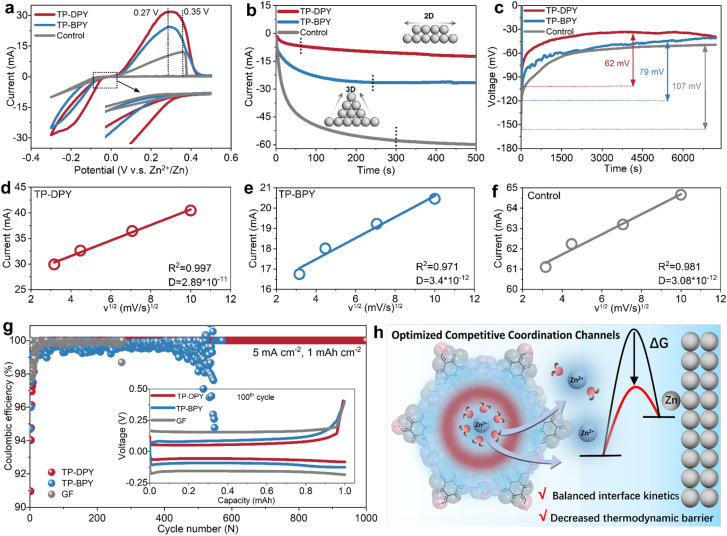
Electrochemical interfacial behavior of Zn electrodes in aqueous electrolyte regulated by pyridine COF membranes. (a) CV curves, (b) CA curves at a bias potential of −150 mV, and (c) CP curves at a current density of 1 mA cm^−2^. Peak current *vs.* the square root of scan rate plots for Zn‖Cu cells paired with (d) TP-DPY membrane, (e) TP-BPY membrane, and (f) bulk electrolyte. (g) Coulombic efficiency of Zn‖Cu cells at a current density of 5 mA cm^−2^; the inset shows the 100th charge–discharge profiles of Zn‖Cu cells with the two COF membranes and bulk electrolyte. (h) Schematic of pyridinyl COF membranes promoting simultaneous zinc ion transport and desolvation with reduced nucleation barriers and balanced interfacial kinetics on the zinc anode.

The electrochemical interfacial behavior of Zn metal electrodes regulated by pyridine COF membranes was systematically investigated across various electrochemical devices, with glass fiber (GF) separators as the control group. Cyclic voltammetry (CV) analysis within Zn‖Cu asymmetric cells demonstrates that TP-DPY and TP-BPY membranes yield lower stripping peak potentials, enhanced peak current densities, and more positive plating potentials relative to the control sample (Fig. 3a). This behavior indicates reduced polarization and lower kinetic overpotentials for zinc stripping and plating, reflecting more efficient interfacial charge transfer and enhanced reaction reversibility enabled by the COF membranes. Comparison between the two COF membranes reveals that the TP-DPY-paired electrode exhibits higher stripping/plating currents, implying faster interfacial kinetics and more efficient zinc redox processes under identical polarization conditions. In chronoamperometry (CA) measurements, as shown in Fig. 3b and S20, cells assembled with COF membranes display smaller and more stable current responses than the control counterpart, indicating a reduced instantaneous reaction rate and a more regulated ion reduction process at the electrode interface. This behavior reflects suppressed current fluctuations and mitigated concentration polarization of the zinc electrode by COF membranes, suggesting that the deposition process proceeds through controlled nucleation and steady growth rather than rapid, unstable interfacial reactions. Simultaneously, chronopotentiometry (CP) measurements reveal that the zinc electrode assembled with pyridinyl COF membranes exhibits a significantly lower nucleation overpotential and a more stable voltage plateau, indicating a reduced thermodynamic barrier for nucleation and facilitating uniform zinc deposition (Fig. 3c).

To further investigate ion diffusion kinetics at the electrode surface mediated by the two different COF membranes, we measured the Zn^2+^ diffusion coefficients (*D*) in Zn–Cu cells assembled with the membranes (Fig. 3d–f and S21).^30^ The TP-DPY-assembled cell exhibited a markedly higher diffusion coefficient (2.89 × 10^−11^ cm^2^ s^−1^) than the TP-BPY membrane (3.4 × 10^−12^ cm^2^ s^−1^) and the control cell (3.08 × 10^−12^ cm^2^ s^−1^). This result indicates that the mono-pyridinyl COF membrane can substantially enhance interfacial ion mobility. The partial desolvation of Zn^2+^ induced by the COF membranes reduces the reorganization energy for electron transfer, thereby lowering the kinetic barrier for reduction. At the same time, the combination of efficient ion transport and the regulated local hydration level at the interface reshapes the free energy landscape for nucleation, decreasing the thermodynamic barrier for metal deposition. These features demonstrate that the COF membranes, particularly TP-DPY, govern both the kinetic and thermodynamic aspects of zinc electrodeposition, enabling faster, energetically favorable, and controlled electrochemical deposition at the electrode surface. As a result, the TP-DPY-based cell retained nearly 100% coulombic efficiency over 1000 cycles of stripping/plating on the Cu substrate, whereas the TP-BPY-based cell exhibited significant coulombic efficiency fluctuations after 500 cycles, reflecting pronounced parasitic reactions and unstable zinc deposition, and short-circuited shortly thereafter (Fig. 3g). In comparison, the control cell failed within 300 cycles. We collected XRD patterns of zinc deposited on the copper substrate after the cycling test (Fig. S22). Compared with the control sample, which exhibits clear diffraction peaks corresponding to side products Zn_4_SO_4_(OH)_6_·*x*H_2_O, no such peaks were observed on the Cu electrode paired with the two COF membranes. This indicates that the COF membranes can effectively suppress the parasitic reaction and the formation of zinc surface byproducts and benefit more uniform and stable metallic zinc deposition. Through a series of the above electrochemical and structural characterization studies, it can be confirmed that pyridinyl COF membranes, particularly TP-DPY, enhance interfacial Zn^2+^ transport, effectively balancing surface diffusion and reaction kinetics at the electrode (Fig. 3h). Simultaneously, COF-mediated partial desolvation lowers the reorganization energy and nucleation free energy, reducing thermodynamic barriers. This dual optimization of kinetics and thermodynamics enhances interfacial reactivity while suppressing parasitic side reactions. The combination of rapid ion conduction with reduced hydration ensures a controlled and stable deposition process, ultimately resulting in uniform, energetically favorable, and highly reversible electrochemical stripping/plating over extended cycling.^31–33^

**Fig. 4:**
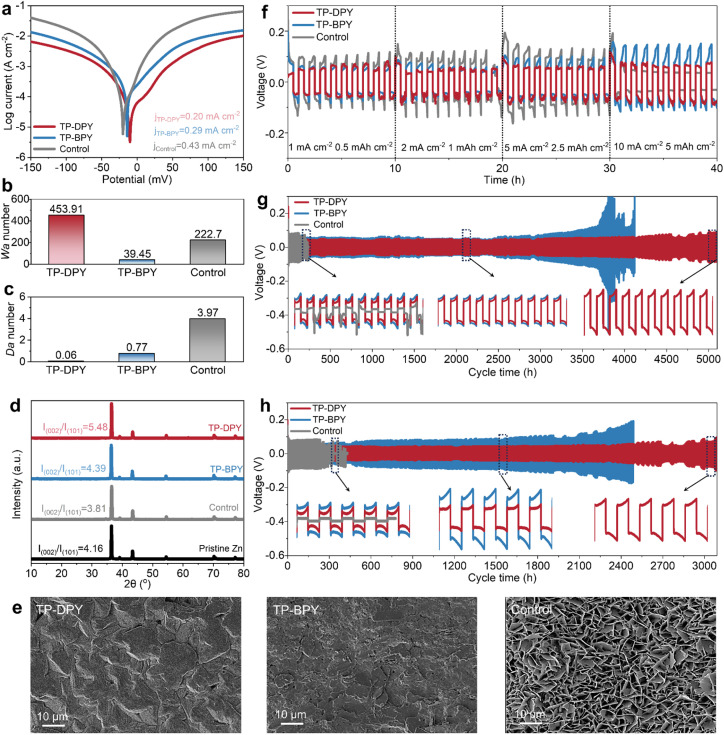
Electrochemical stability of Zn electrodes in aqueous electrolyte regulated by pyridine COF membranes. (a) Tafel plots, (b) *W*_a_ numbers, and (c) *D*_a_ numbers of Zn‖Zn cells with different membranes. (d) XRD patterns and (e) SEM images of Zn electrodes after 100 cycles. (f) Rate performance of Zn‖Zn cells with different separators. Charge–discharge profiles of Zn‖Zn symmetric cells with different membranes at a current density of (g) 1 mA cm^−2^ with an areal capacity of 1 mAh cm^−2^ and (h) 5 mA cm^−2^ with an areal capacity of 5 mAh cm^−2^.

We systematically evaluated the electrochemical stability of zinc electrodes in aqueous electrolytes under the regulation of the two pyridinyl COF membranes. First, the Tafel curves of Zn‖Zn symmetric cells paired with different membranes were collected to reveal the electrochemical corrosion behavior and deposition stability of the zinc electrode in ZnSO_4_ aqueous electrolyte. As shown in Fig. 4a, the Tafel plots of cells assembled with TP-DPY, TP-BPY, and conventional GF separators as the control sample show that the corrosion current density is significantly reduced with both pyridine-based COF separators. Specifically, cells assembled with TP-DPY and TP-BPY separators exhibit markedly reduced corrosion currents down to 0.20 and 0.29 mA cm^−2^, respectively, compared to 0.43 mA cm^−2^ for the GF-assembled cell. Moreover, the TP-DPY-based cell shows a more negative corrosion potential. This indicates that pyridinyl COF membranes effectively suppress zinc electrode corrosion by slowing spontaneous dissolution and enhancing interfacial stability. The more negative corrosion potential further reflects increased thermodynamic resistance to anodic dissolution. Together, these results demonstrate that the COF membranes not only improve interfacial charge-transfer kinetics but also provide a more uniform and controlled Zn^2+^ deposition environment, thereby collectively enhancing the long-term stability of the electrode.

We further utilized the Damköhler (*D*_a_) number and Wagner (*W*_a_) number to investigate the impact of pyridine-based COF membranes on zinc deposition and dendrite growth behavior, which is crucial for the cycling stability of zinc electrodes. The *D*_a_ and *W*_a_ numbers evaluate the competition between diffusion and electrochemical reaction rates, respectively.^34–36^ As shown in Fig. 4b and c, the cell assembled with the TP-DPY COF membrane exhibits a higher *W*_a_ number and a smaller *D*_a_ number compared to the TP-BPY and control cells. This combination indicates that zinc deposition paired with the TP-DPY COF membrane is primarily diffusion-controlled rather than reaction-controlled. From a kinetic perspective, the deposition process is limited by Zn^2+^ diffusion rather than electron-transfer kinetics, suggesting that ion migration is governed by diffusion rates. From a thermodynamic perspective, the TP-DPY COF membrane minimizes the local supersaturation during nucleation. This effect can theoretically induce uniform and dense zinc deposition. This can be confirmed by X-ray diffraction (XRD) and scanning electron microscopy (SEM) characterization. XRD patterns show that the pyridine-based COF membrane does not significantly change the crystallographic orientation of the zinc crystal (Fig. 4d). SEM images clearly show that the TP-DPY COF membrane leads to more uniform and denser and smooth zinc deposition compared to that of the control electrode and TP-BPY COF membranes (Fig. 4e and S25). In addition, X-ray photoelectron spectroscopy (XPS) characterization was performed on zinc electrodes from Zn‖Zn symmetric cells assembled with different membranes after long-term cycling to investigate the Zn chemical states on their surfaces (Fig. S24). The results show that electrodes in cells with the TP-DPY COF membrane predominantly consist of metallic Zn; those with the TP-BPY membrane exhibit a minor divalent Zn (Zn^2+^) signal, and those with the GF separator display a significant Zn^2+^ signal. Based on these observations, the formation of insoluble zinc byproducts can be inferred, indicating that COF membranes, particularly TP-DPY, effectively suppress parasitic reactions during cycling.

The cycling stability of zinc electrodes regulated by COF membranes was further evaluated using Zn‖Zn symmetric cells. Rate performance tests indicate that with increasing current density, cells assembled with the TP-BPY COF membrane and the GF separator exhibit pronounced polarization. At a high current density of 10 mA cm^−2^, cells with the GF separator even experience short-circuit failure (Fig. 4f). In contrast, cells incorporating the TP-DPY COF membrane retain stable voltage profiles across all tested current densities without any signs of short-circuiting. During long-term cycling at a standard 1 mA cm^−2^ with an aerial capacity of 1 mAh cm^−2^, the TP-DPY-paired cell operates stably for up to 5000 hours, far exceeding the lifespan of cells with GF (<300 hours) and the TP-BPY (∼4000 hours) membrane (Fig. 4g). Even at an increased current density of 5 mA cm^−2^, the TP-DPY-assembled cell continues to cycle steadily for up to 3000 hours (Fig. 4h), demonstrating significantly improved cycling stability compared to that of GF and TP-BPY membranes. In fact, the membrane developed in this work, particularly the TP-DPY membrane, enables zinc electrodes to achieve cycling lifetimes that surpass those of most previously reported porous membranes used in AZBs (Table S4), highlighting the superior performance of competitive coordination design. In addition, we evaluated the deep stripping/plating performance of Zn‖Zn symmetric cells assembled with different membranes, which is a stringent test of the stability of electrodes with the ability to suppress parasitic reactions and dendrite growth. Under high depth-of-discharge conditions (DOD = 50%), the TP-DPY-based cell retained stable cycling throughout the entire testing period (Fig. S26). In contrast, the TP-BPY-based cell failed within fewer than ten cycles, while the cell using the GF separator showed no stable deep stripping and plating process. These results clearly demonstrate that the rapid hydrated zinc ion conduction and partial desolvation facilitated by pyridinyl COF membranes can substantially enhance the electrochemical cycling stability of zinc metal electrodes in aqueous electrolytes.

**Fig. 5:**
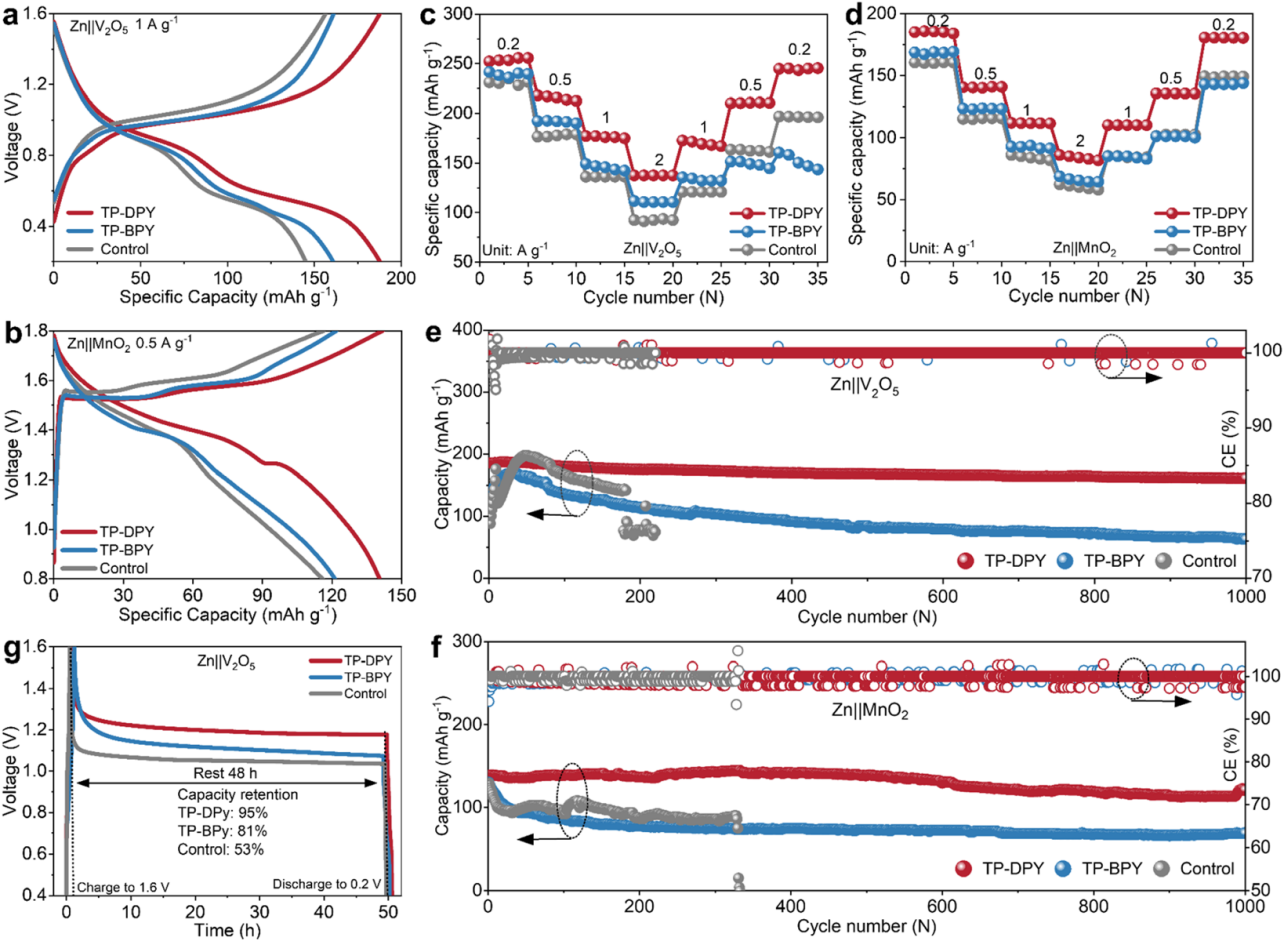
Electrochemical performance evaluation of pyridine COF membranes in full battery systems. (a and b) Charge–discharge curves and (c and d) rate performance of Zn‖V_2_O_5_ and Zn‖MnO_2_ full batteries paired with different membranes. (e) Discharge capacity and Coulombic efficiency of long-term cycling of Zn‖V_2_O_5_ full batteries at 1 A g^−1^ and (f) Zn‖MnO_2_ full batteries at 0.5 A g^−1^. (g) Self-discharge profiles of Zn‖V_2_O_5_ full batteries with different membranes.

The electrochemical performance of the two pyridine COF membranes was further evaluated in full batteries by using commercial V_2_O_5_ and MnO_2_ as cathode materials, respectively. In parallel, batteries paired with the conventional GF separator were also examined as control samples for comparative analysis. As is well known, V_2_O_5_ and MnO_2_ store energy in aqueous zinc-ion batteries through reversible Zn^2+^ insertion and extraction accompanied by reversible redox transitions of the metal centers,^37^ which places stringent requirements on electrolyte ions to exhibit high mobility and a reduced solvation shell so as to support rapid diffusion, suppress polarization, and retain the structural stability of the cathode. Fig. 5a and b present the charge–discharge profiles of Zn‖V_2_O_5_ and Zn‖MnO_2_ full batteries assembled with different COF membranes. Both full batteries incorporating the TP-DPY membrane exhibit a smaller voltage gap between the charge and discharge plateaus, indicating reduced polarization and more efficient interfacial redox kinetics. At identical current densities, these TP-DPY-based full batteries also deliver higher specific capacities, confirming that the rapid Zn^2+^ transport and partial desolvation facilitated by the TP-DPY membrane enhance intercalation kinetics. In addition, rate performance tests show that, with increasing current density, the capacity decay of TP-DPY-assembled full batteries is significantly less pronounced than that of TP-BPY-based and control batteries. When the current density is subsequently reduced, TP-BPY-assembled batteries nearly recover their original capacity, indicating that their capacity loss under high-rate conditions is primarily limited by kinetic constraints rather than irreversible structural degradation (Fig. 5c and d). This performance can be attributed to the rapid Zn^2+^ transport and partial desolvation provided by the TP-DPY membrane, which facilitate efficient ion migration, minimize local concentration gradients, and retain uniform intercalation throughout the cathode structure.

The cycling charge–discharge tests reveal that the full batteries with the GF separator experience an obvious short-circuit failure after fewer than 300 cycles (Fig. 5e and f), which is consistent with previously reported performance for similar systems. The two full batteries assembled with the TP-BPY membrane did not experience short-circuiting, yet their specific capacities showed significant decay after 1000 cycles, falling to less than 50% of the initial values. By comparison, the TP-DPY membrane enables the two full batteries to retain highly stable specific capacities over 1000 cycles, retaining more than 85% of their initial values, while the Coulombic efficiency remains consistently around 100% with negligible fluctuations. We also characterized the cathode materials after the cycling test. Under the same tested conditions, SEM, EDS, and XRD analyses show that both cathodes retain their structural integrity (Fig. S29 and 30). The separator has minimal impact on their morphology and crystal structure. These results indicate that the enhancement of cycle stability observed in full batteries primarily arises from the effects of the COF membranes on the Zn anode and the electrolyte. The post-cycling analysis of the separator after long-term cycling revealed that it retained uniform thickness and porosity (Fig. S31–33) and chemical structural integrity (Fig. S34), indicating excellent structural stability during operation.

Moreover, we evaluated the self-discharge behavior of full batteries assembled with the two pyridinyl COF membranes, which is closely related to the spontaneous dissolution of the zinc anode in aqueous electrolytes. The self-discharge test was conducted by first fully charging the batteries to a specified capacity, followed by a 48-hour resting period under open-circuit conditions, and then discharging them to measure the remaining capacity (Fig. 5g). The results show that the full battery with the TP-DPY membrane retains more than 90% of its capacity (Fig. 5g and S35), significantly higher than those with the TP-BPY and GF separators, demonstrating the excellent self-discharge suppression capability of TP-DPY. This is mainly attributed to the partial desolvation of Zn^2+^ induced by COF membranes, which significantly suppresses self-discharge through both kinetic and thermodynamic mechanisms. Partially desolvated Zn^2+^ carries fewer water molecules, reducing the concentration of reactive hydrated ions at the zinc surface and accelerating ion diffusion away from the electrode, thereby lowering local overpotentials and mitigating spontaneous dissolution. Thermodynamically, the COF-mediated coordination environment stabilizes the desolvated Zn^2+^ and increases the energy barrier for electron transfer and zinc oxidation, thus decreasing the driving force for self-discharge under open-circuit conditions.

## Conclusion

In summary, this work has demonstrated that mono-pyridinyl-functionalized COF membranes exhibit rapid transport of hydrated ions while promoting their partial desolvation. By a combination of theoretical calculations and spectroscopic characterization, we have confirmed that this unique ion transport behavior originates from a soft Lewis acid-induced competitive coordination mechanism within the nanochannels of pyridinyl COF membranes. This membrane-mediated rapid transport of desolvated hydrated ions can significantly enhance the reversibility and cycling stability of aqueous electrochemical energy storage devices. We performed a systematic proof-of-concept evaluation of the electrochemical performance of pyridinyl COF membranes using aqueous zinc-ion batteries as a model system. Electrochemical measurements indicate that this combination of rapid ion conduction and efficient desolvation enhances interfacial Zn^2+^ diffusion, lowers nucleation overpotentials, and regulates surface deposition kinetics, thereby promoting uniform zinc growth and effectively suppressing dendrite formation. Zinc metal electrodes paired with mono-pyridine COF membranes could retain stable stripping/plating over 2000 cycles in a 2 M ZnSO_4_ electrolyte without any additives, far exceeding the performance of currently employed aqueous battery separators. Full battery tests using MnO_2_ and V_2_O_5_ cathodes demonstrate that the mono-pyridine COF membrane enables the batteries to retain more than 85% of their initial capacity after 1000 charge–discharge cycles. These results highlight the potential of COF membranes as a platform for precise regulation of ion solvation and transport, offering a versatile strategy to enhance the efficiency, stability, and safety of next-generation aqueous electrochemical energy storage devices.

## Author contributions

S. Zhou proposed and supervised the project, conceptualized and designed the study, analyzed the data, and wrote the initial manuscript together with X. Li. X. Li carried out most of the experiments and theoretical calculations. J. Liu, Y. Zhang, Z. Dai, and Z. Tan were responsible for material characterization. All authors participated in discussing the results and preparing the final manuscript.

## Conflicts of interest

The authors declare no competing interests.

## Supplementary Material

SC-OLF-D6SC01290F-s001

## Data Availability

The data supporting this article have been included as part of the supplementary information (SI). Supplementary information is available. See DOI: https://doi.org/10.1039/d6sc01290f.
