# Supplementary material for: Fast hydrated-ion transport and desolvation in pyridinyl COF membranes via competitive coordination
Source: Chem Sci. 2026 Apr 13;17(21):10658–68. doi: 10.1039/d6sc01290f (PMC13093751; doi:10.1039/d6sc01290f)
Supplement: SC-017-D6SC01290F-s001 [file SC-017-D6SC01290F-s001.pdf]

Supplementary Information (SI) for Chemical Science.
This journal is © The Royal Society of Chemistry 2026

Supplementary Information

Fast Hydrated-Ion Transport and Desolvation in Pyridinyl COF Membranes via Competitive Coordination

Xuan Li,[1] Jiaxin Liu, [1] Yilin Zhang,[1] Zhixiang Dai,[1] Zihan Tan,[1] Hongli Yang,[1] Chao Xu,[2] Maria Strømme,[2] Shengyang Zhou, [1,] Zhong-Ming Li[1]*

[1]College of Materials Science and Engineering, College of Polymer Science and Engineering, State Key Laboratory of Advanced Polymer Materials, Sichuan University, Chengdu 610065, China

[2]Department of Materials Science and Engineering, The Ångström Laboratory, Uppsala University, Uppsala 752 37, Sweden

[*]E-mail: shengyang.zhou@scu.edu.cn

Experimental Section

Materials. Zinc sulfate heptahydrate ($ZnSO_4 \cdot 7H_2O$, AR, Aladdin), Manganese sulfate monohydrate ($MnSO_4 \cdot H_2O$, AR, Aladdin), zinc foil (50 μm, Olegeeino), cooper foil (50 μm, Olegeeino), glass fiber (Olegeeino), cellulose (FMC Biopolymer, U.S.A.), 2,4,6-Triformylphloroglucinol (TP, Aladdin), 2,6-Diaminopyridine (DPY, Aladdin), 2,2′-bipyridine-5,5′-diamine (BPY, Aladdin) and acetic acid (AR, Aladdin) were used without any treatment. MnO_2 and V_2O_5 cathodes were provided by Future (Jilin) Materials Tech. Co. Ltd. The water used in the study was ultra-pure (18.2 MΩ · cm), produced by an ultra-pure water system (Shanghai Lichen Instrument Technology Co., Ltd.).

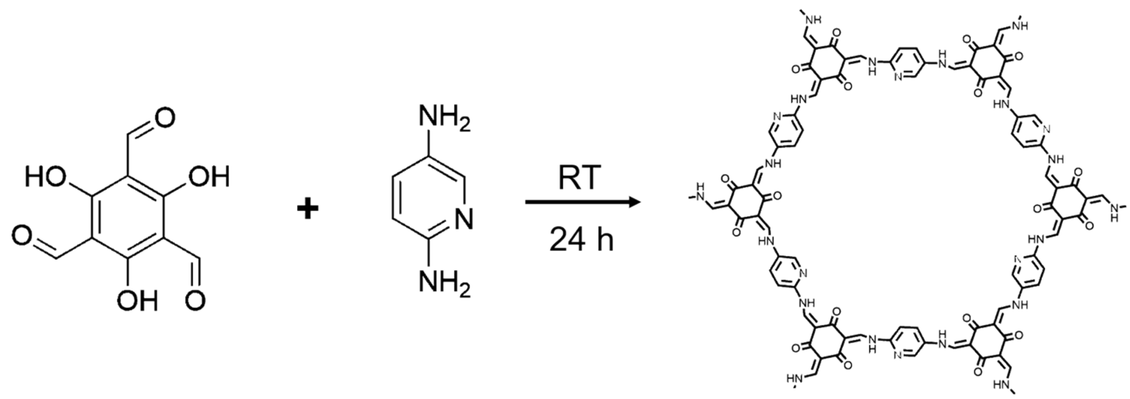

Figure S1. Synthesis of TP-DPY COFs.

Synthesis of TP-DPY COFs. 129 mg (0.6 mmol) TP and 4 mL aqueous acetic acid solution (6 M) were added into a 20 mL glass vial. The mixture was dissolved by ultrasound. Meanwhile, 109 mg (0.9 mmol) DPY was dissolved in 4 mL DI water assisted by sonication and the solution was dropwise added into the glass vial. The mixture was placed on hold at room temperature for 24 h (Figure S1).

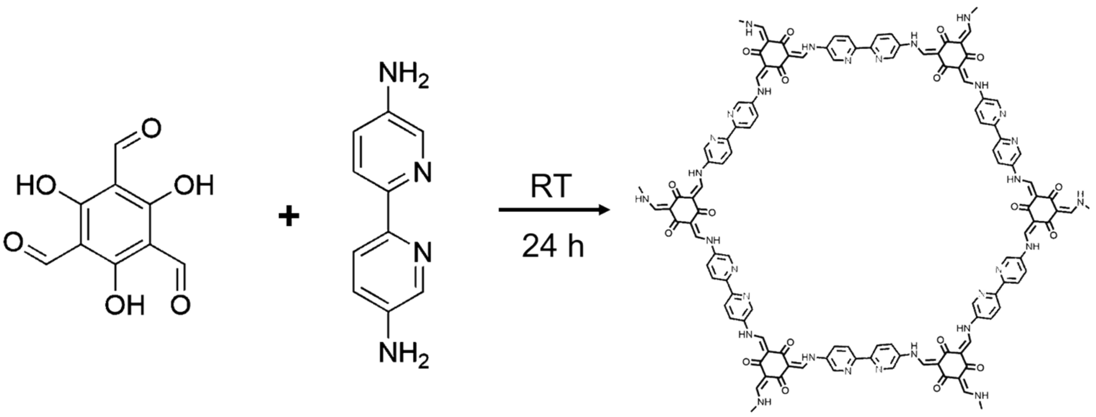

Figure S2. Synthesis of TP-BPY COFs.

Synthesis of TP-BPY COFs. 129 mg (0.6 mmol) TP and 4 mL aqueous acetic acid solution (6 M) were added into a 20 mL glass vial. The mixture was dissolved by ultrasound. Meanwhile, 171 mg (0.9 mmol) BPY was dissolved in 4 mL DI water assisted by sonication and the solution was dropwise added into the glass vial. The mixture was placed on hold at room temperature for (Figure S2).

Preparation of TP-DPY (TP-BPY) Membranes. 5 mL TP-DPY (TP-BPY) solution was added into 40 mL cellulose aqueous solution (2.5 mg mL^{-1}), then sonicated for 10 minutes to make the solution blend uniformly. Half of the above-mixed solution and 20 mL of pure cellulose solution (2.5 mg mL^{-1}) were poured into the vacuum extraction filter, successively. Following that, the remaining half-mixed solution was poured into the filter to obtain the separator with a sandwich structure. The TP-DPY (TP-BPY) separator could be finally obtained after drying overnight in the oven at 60 °C. Finally, the separator was cut into discs with a diameter of 16 mm. The thickness of prepared COF membranes was controlled at 30-90 μm. The mass loading of COFs is determined to be 0.2 mg cm^{-2} with a porosity of around 30%.

Materials Characterization. The X-ray diffraction (XRD) profiles were collected by an X-ray diffractometer with Cu K_{α} radiation (DX-2700B, HAOYUAN Instrument). The scanning electron microscopy (SEM) images were collected by a Nova NanoSEM (450, FEI). X-ray spectroscopy (EDS) mapping images were collected by a Nova NanoSEM (450, FEI). Wide-angle X-ray scattering (WAXS) patterns were acquired on beamline 16B of the Shanghai Synchrotron Radiation Facility (Shanghai, China). A nitrogen adsorption-desorption analyzer (Ankersmid Belsorp-Max) was used to investigate the distribution of the separator pore size and diameter. The mechanical and wettability properties of separators were tested by a universal test machine (INSTRON 5967 Universal Testing Systems) and a contact angle measurement device (Kruss DSA30S), respectively. H magnetic resonance imaging (NMR spectroscopy) was performed on Bruker AV II-600 MHz. Fourier transform infrared spectroscopy (FTIR) was performed by using a Thermo Scientific Nicolet 6700.

Electrochemical Characterization. The commercial Zn foil (50 μm) and Cu foil (50 μm) were cut into discs with a diameter of 12 mm. Zn||Zn, Zn||Cu cells were assembled using CR2032 coin-type cells by adding 100 μL 2 M $ZnSO_4$ electrolyte at room temperature. Zn||MnO_2 full batteries were assembled using CR2032 coin-type cells by adding 100 μL 2 M $ZnSO_4$ / 0.2 M $MnSO_4$ electrolyte at room temperature. The voltage window for Zn||MnO_2 full battery cycling test was set from 0.8 V to 1.8 V. Zn||V_2O_5 full batteries were assembled using CR2032 coin-type cells by adding 100 μL 2 M $ZnSO_4$ electrolyte at room temperature. The voltage window for Zn||V_2O_5 full battery cycling test was set from 0.2 V to 1.6 V. Electrochemical measurements, including

cyclic voltammetry (CV), chronoamperometry (CA), electrochemical impedance spectroscopy (EIS), Tafel plots, linear sweep voltammetry (LSV) and chronopotentiometry (CP) were conducted using an electrochemical workstation (CHI660E, Chenhua). EIS tests were conducted over a frequency range of 0.05 to 10^5 Hz. Tafel tests were performed between -0.2 V and 0.2 V under 1 mV s^{-1}· CV tests for Zn||Cu half cells were carried out between -0.2 V and 0.5 V at 1 mV s^{-1}. CV test for Zn||V_2O_5 full cell was carried out between 0.2 V and 1.6 V at 1 mV s^{-1}. CV test for Zn||MnO_2 full cell was carried out between 0.8 V and 1.8 V at 1 mV s^{-1}. The battery tests were implemented using a Neware battery system (CT-4008T).

Ionic Conductivity Measurements. The ionic conductivities (σ) of the separators were measured using two stainless-steel electrodes. The σ can be calculated through the following equation[1]:

$$\sigma = \frac{L}{RS}$$

where L is the thickness of the separator, R is the resistance based on the EIS test, and S is the contact area between the separator and stainless-steel electrode. The parameters of the different separators to calculate ionic conductivity are listed in Table S1.

Table S1. The parameters to calculate ionic conductivity.

**Separator**	**Thickness** (μm)	**R** (Ω)	**Ionic conductivity** (mS cm^{-1})
TP-DPY	60	0.33	10.2
TP-BPY	60	0.36	9.4
GF	220	5.1	2.4

Zn^{2+} Transfer Number Measurements. Zn^{2+} transfer numbers ($t_{Zn^{2+}}$) of different

separators were measured by assembling Zn||Zn symmetric cells based on the following calculation equation[1]:

$$t_{Zn^{2+}} = \frac{I_S(\Delta V - I_0 R_0)}{I_0(\Delta V - I_S R_S)}$$

where ΔV is the constant polarization voltage (50 mV), I_0 and I_S are the initial current and the steady-state current based on CA test, respectively. R_0 and R_s are the initial resistance and the stable resistance based on EIS test, respectively.

Activation Energy Measurements. The desolvation activation energy (E_a) of hydrated Zn^{2+} was measured using Zn||Zn cells at different temperatures. The E_a can be calculated through the following equation[2]:

$$\frac{1}{R_{ct}} = Aexp(\frac{-E_a}{RT})$$

where R_{ct} is the resistance obtained from the EIS spectra, R is the gas constant, and T is the thermodynamic temperature.

Measurement of Zn^{2+} Diffusion Coefficient. The diffusion coefficient of Zn^{2+} (D) was measured using Zn||Cu cells. Under different scan rates, CV curves are scanned in the same voltage range. The D can be calculated through the following equation[3]:

$$I_p = 0.4463nFAC_x V^{1/2}\sqrt{\frac{nFD_X}{RT}}$$

where I_p is the peak current, n is the number of electrons gained and lost in the electrode reaction, F is the Faraday constant, A is the contact area between the electrode plate and electrode solution, C_x is the concentration of the Zn^{2+}, V is the scanning rate.

Measurement of D_a and W_a Number. *Damköhler* number (D_a) can be obtained by the

following equation[4]:

$$D_a = \frac{i_0}{i_l}$$

i_0 is the exchange current at the electrode which is obtained from GCD curves, i_l is the diffusion-limited current in the electrolyte which is calculated from:

$$i_l = \frac{4FCD_+}{L}$$

F is the Faraday constant, C is the salt concentration in the electrolyte solution, D_+ is the cation diffusion coefficient, and L is the space between physically separated electrodes.

Wagner number (W_a) can be obtained by the following equation[5]:

$$W_a = \frac{R_{ct}}{R_\Omega}$$

R_{ct} is the charge transfer resistance from the EIS spectra and R_Ω electrolyte resistance from the EIS spectra.

Adsorption Energy Calculations. All calculations were implemented in Materials Studio with the DMol3 code. The Perdew-Burke-Ernzerhof (PBE) functional of the generalized gradient approximation (GGA) was used to calculate the exchange-correlation energy. The DFT semi-core pseudopotentials (DSPPs) method was used to introduce a certain degree of relativistic correction to the atoms and the double numerical plus polarization (DNP) was chosen during the geometry optimization. The convergence tolerances of energy change, maximum force, and maximum displacement were set as 2×10^{-5} Ha, 0.004 Ha $Å^{-1}$, and 0.005 Å, respectively. The adsorption energy

(E_{ads}) of species is calculated by:

$$E_{ads} = E_{(system)} - E_{(catalyst)} - E_{(species)}$$

where $E_{(system)}$, $E_{(catalyst)}$, and $E_{(species)}$ are the total energy of the optimized system with adsorbed species, the isolated catalyst, and species, respectively.

Molecular Dynamics (MD) Simulations. All covalent organic framework (COFs) structures were cleaved along the (001) crystal plane, and a vacuum layer of 15.0 Å was introduced. The Amorphous Cell module in Materials Studio 2020 constructed a three-layer COFs structure. The COFs framework was then packed with water and zinc sulfate molecules at a H_2O: $ZnSO_4$ = 555: 20. Subsequent geometry optimizations and molecular dynamics (MD) simulations were performed using the Forcite module. The energy convergence criterion was set to 2.0×10^{-5} kcal mol^{-1}, and the force convergence threshold was 0.001 kcal mol^{-1} Å^{-1}. The Universal Force Field (UFF) was employed for all simulations, with atomic charges assigned using the charge equilibration (Q_{Eq}) method. Electrostatic and van der Waals interactions were computed using the Ewald summation technique. MD simulations were conducted under NPT ensemble conditions at 298.15 K and 1 atm. A Nosé–Hoover thermostat (damping parameter = 0.1 ps) and a Berendsen barostat (time constant = 1.0 ps) were used for temperature and pressure control, respectively. The system was equilibrated for 10 ns to ensure stability.

Supplementary Results

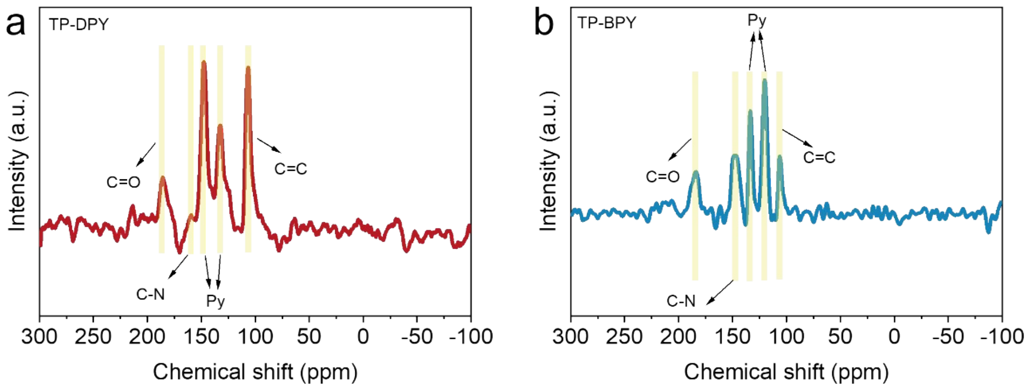

Figure S3. Solid-state ^{13}C NMR spectra of (a) TP-DPY and (b) TP-BPY COF.

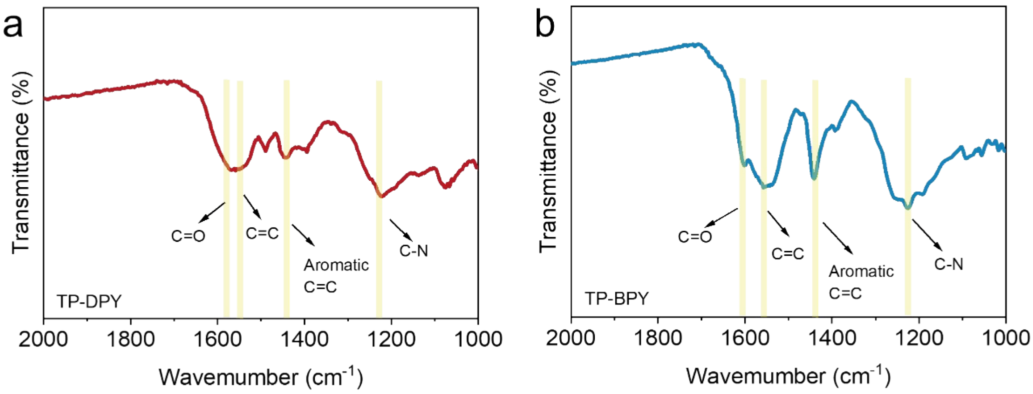

Figure S4. FTIR spectrum of (a) TP-DPY and (b) TP-BPY COF.

a

b

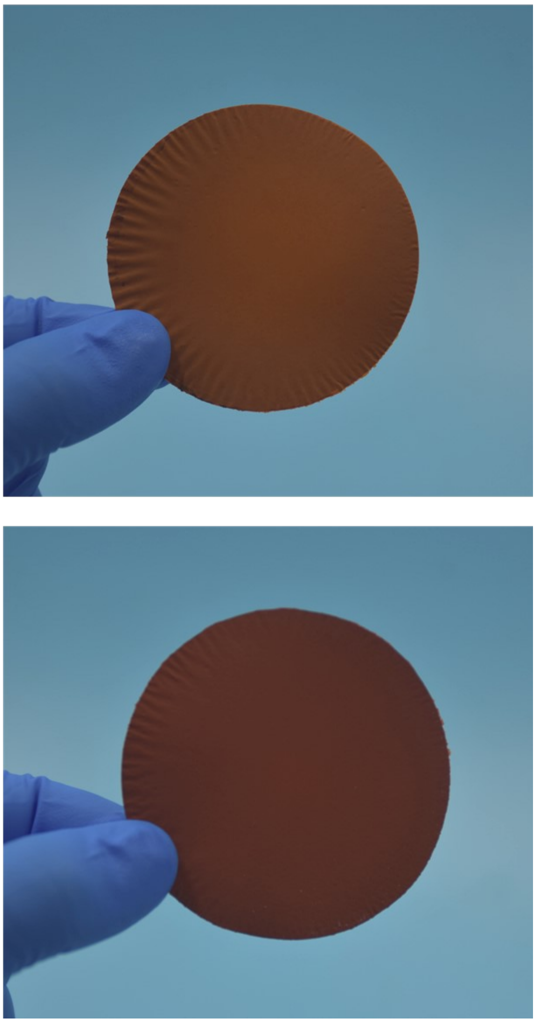

Figure S5. Photo of (a) TP-DPY, (b) TP-BPY membranes.

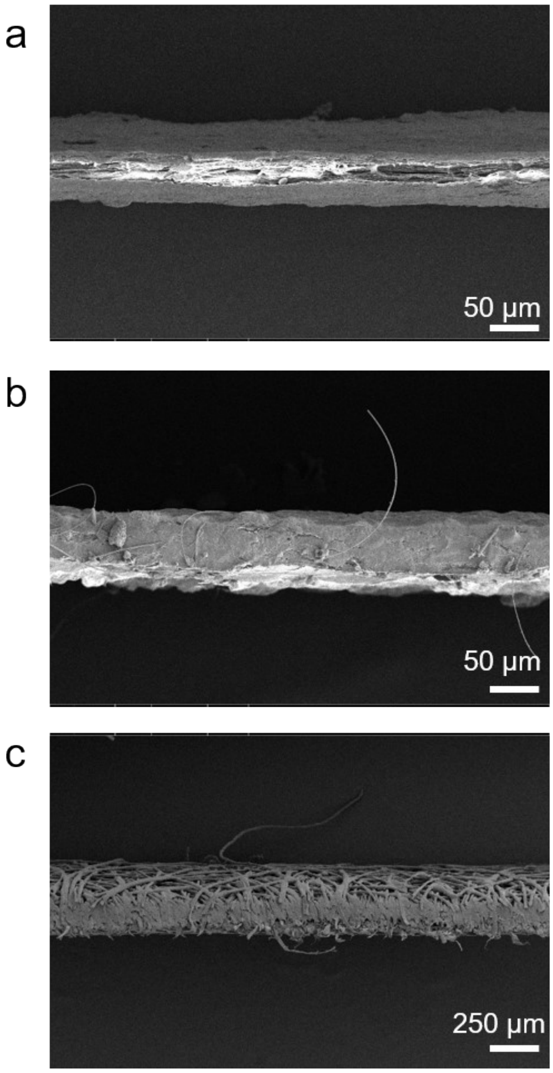

Fig S6. Side-view scanning electron microscopy (SEM) images of (a) TP-DPY, (b) TP-BPY, (c) glass fiber (GF) membranes.

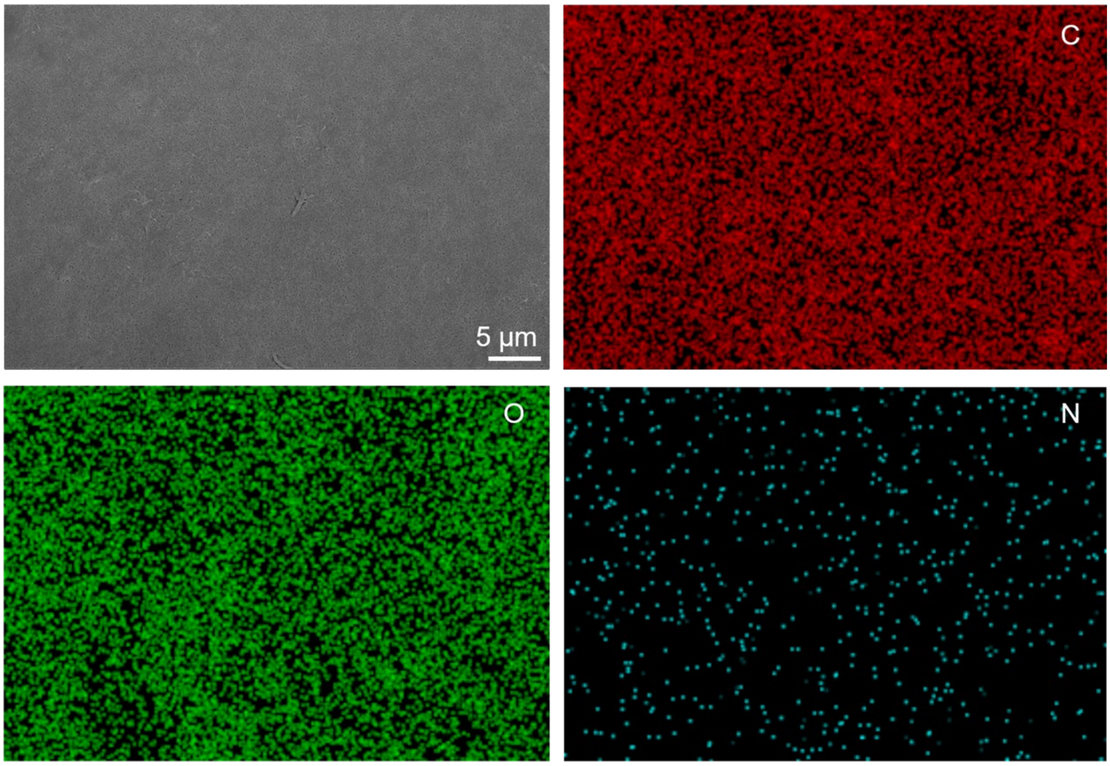

Figure S7. Energy-dispersive X-ray spectroscopy (EDS) mapping images of TP-DPY separator, showing the uniform dispersion of TP-DPY nanoparticles in the membrane.

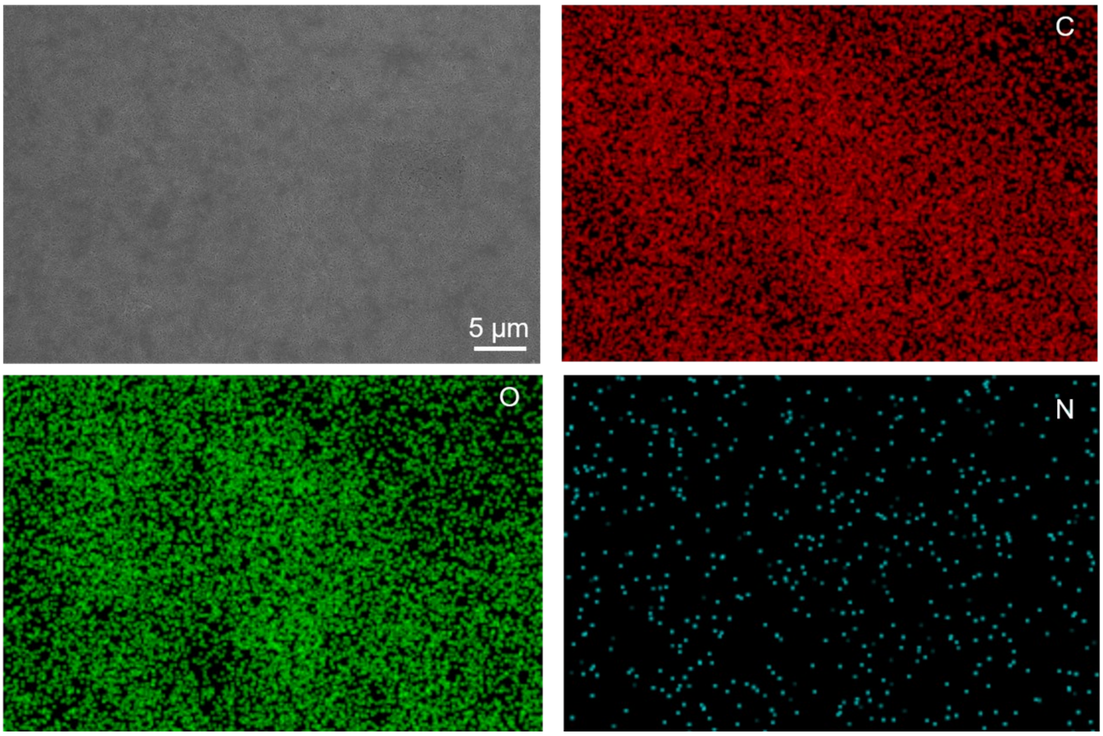

Figure S8. Energy-dispersive X-ray spectroscopy (EDS) mapping images of TP-BPY separator, showing the uniform dispersion of TP-BPY nanoparticles in the membrane.

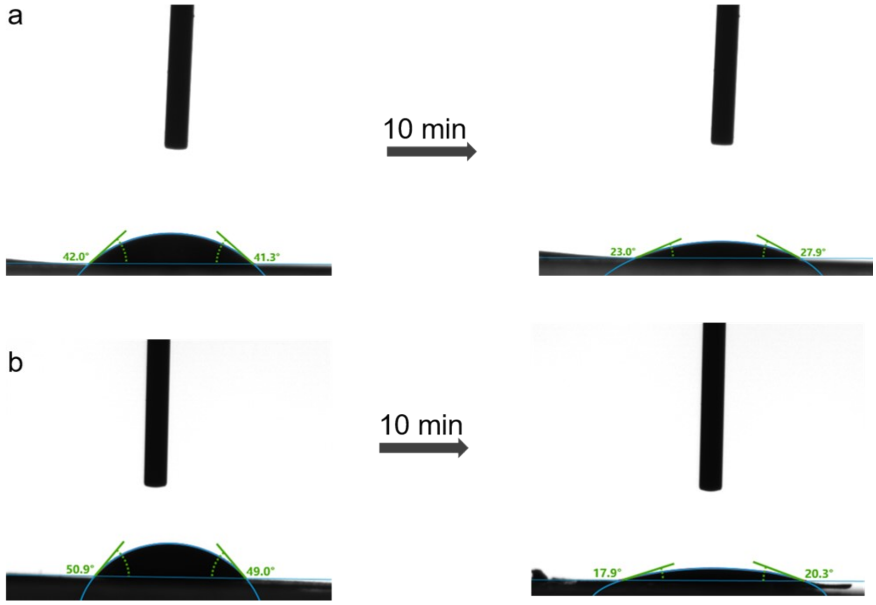

Figure S9. Contact angles test of (a) TP-DPY and (b) TP-BPY membranes with 2 M $ZnSO_4$ electrolyte, suggesting their good wettability.

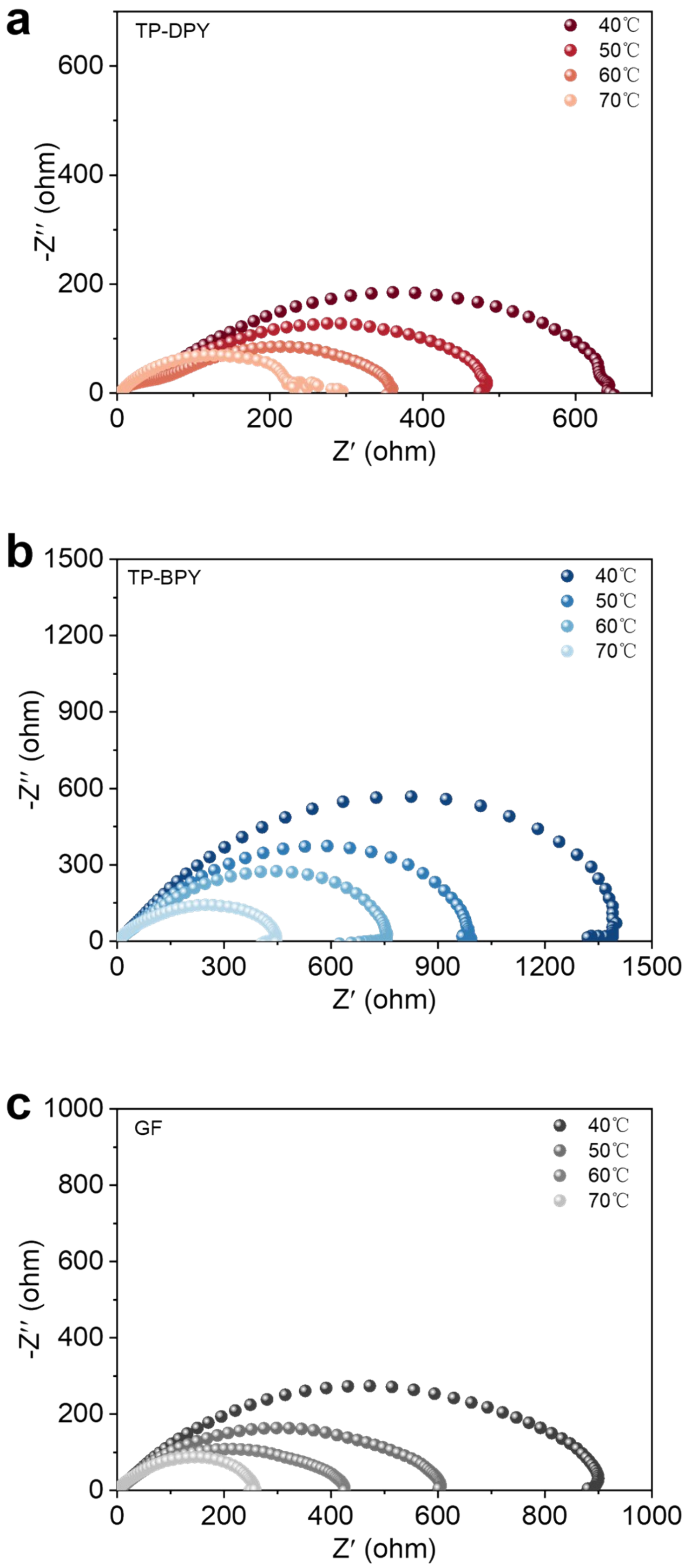

Figure S10. Nyquist plots of Zn||Zn symmetrical cell with (a) TP-DPY, (b) TP-BPY, (c) glass fiber (GF) membranes at different temperatures.

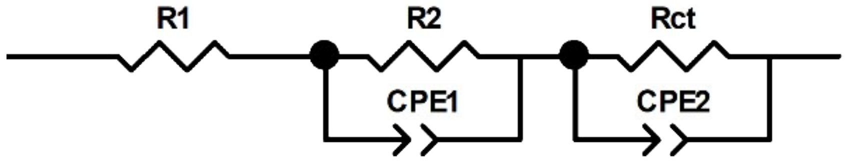

Figure S11. Equivalent circuit model for EIS simulation.

Table S2. The Rct of different membranes collected at different temperatures.

Temperature	TP-DPY	TP-BPY	GF
40 ℃	650.4	1344.0	919.9
50 ℃	483.3	1914.1	560.1
60 ℃	325.8	706.1	399.9
70 ℃	243.5	392.3	230.3

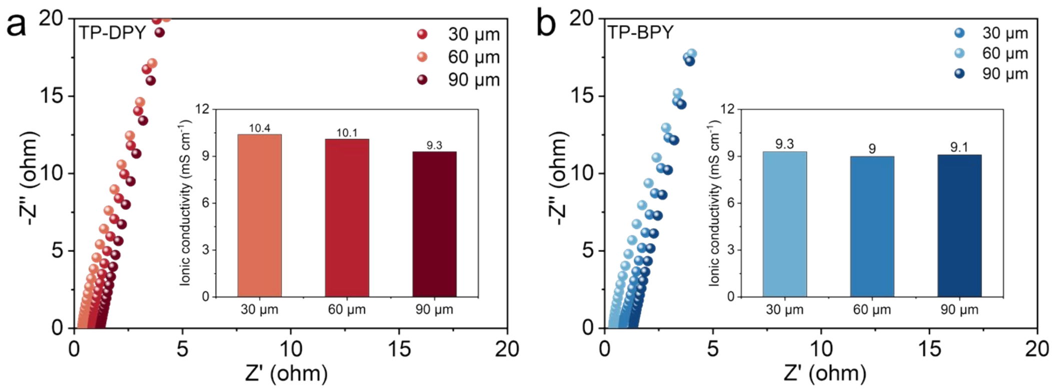

Figure S12. Nyquist plots and calculated ionic conductivity of (a) TP-DPY, (b) TP-BPY membranes with different thickness.

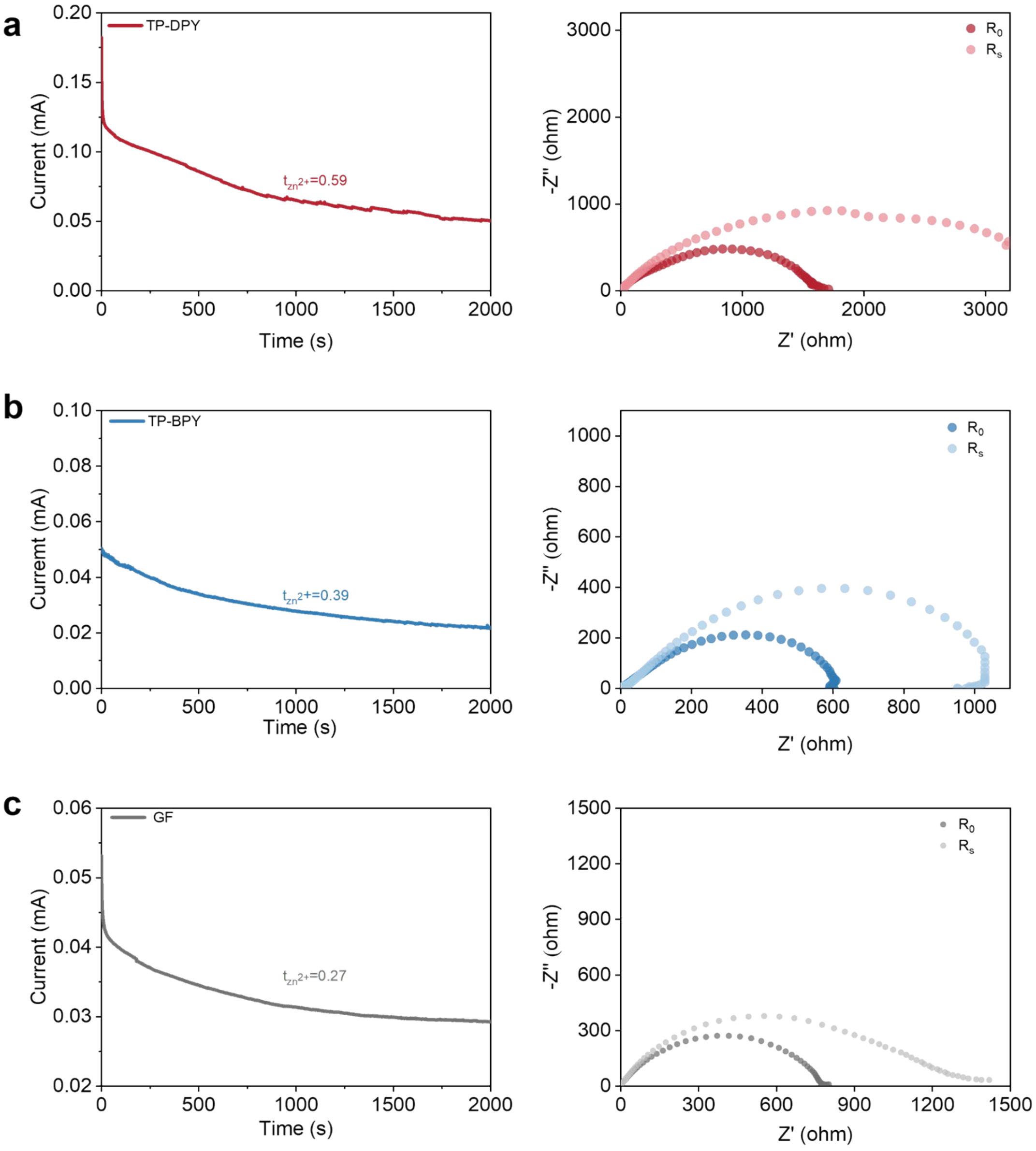

Figure S13. Chronoamperometry (CA) curves and ion transport number of Zn||Zn cells with (a) TP-DPY, (b) TP-BPY, (c) glass fiber (GF) membranes, respectively. The bias voltage was set at 50 mV and the corresponding Nyquist plots were collected at the initial and steady states

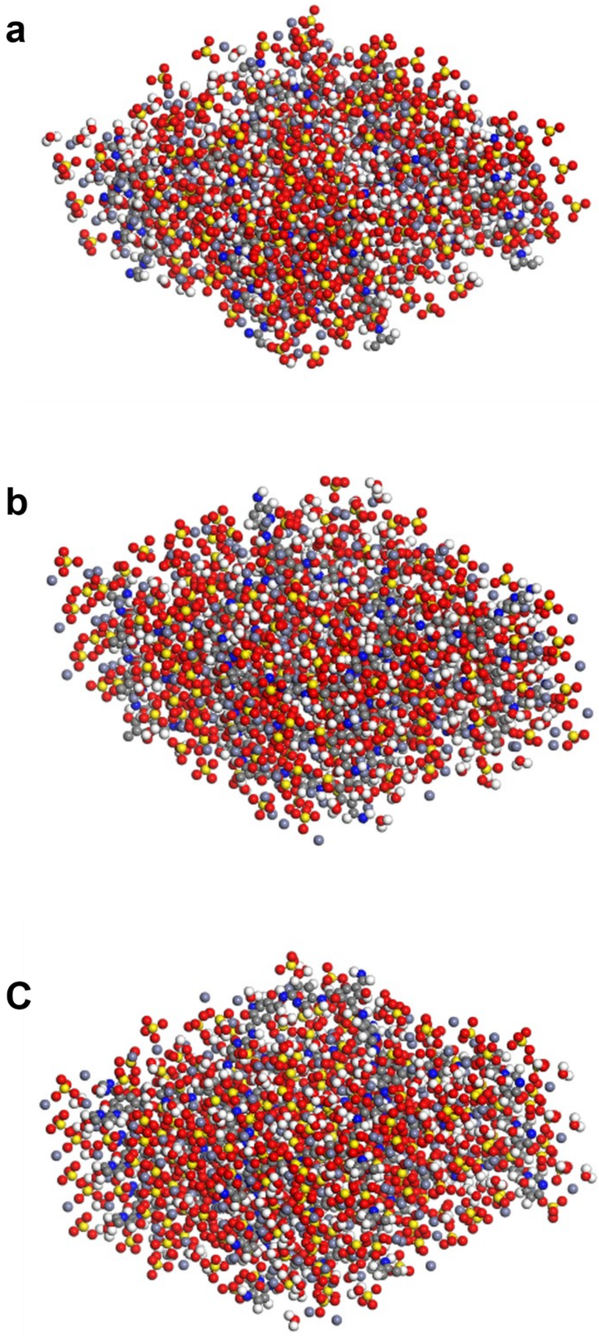

Figure S14. Snapshots from molecular dynamics (MD) simulations of 2 M $ZnSO_4$ with TP-DPY COFs (a) 0 ns, (b) 5 ns, (c) 10 ns.

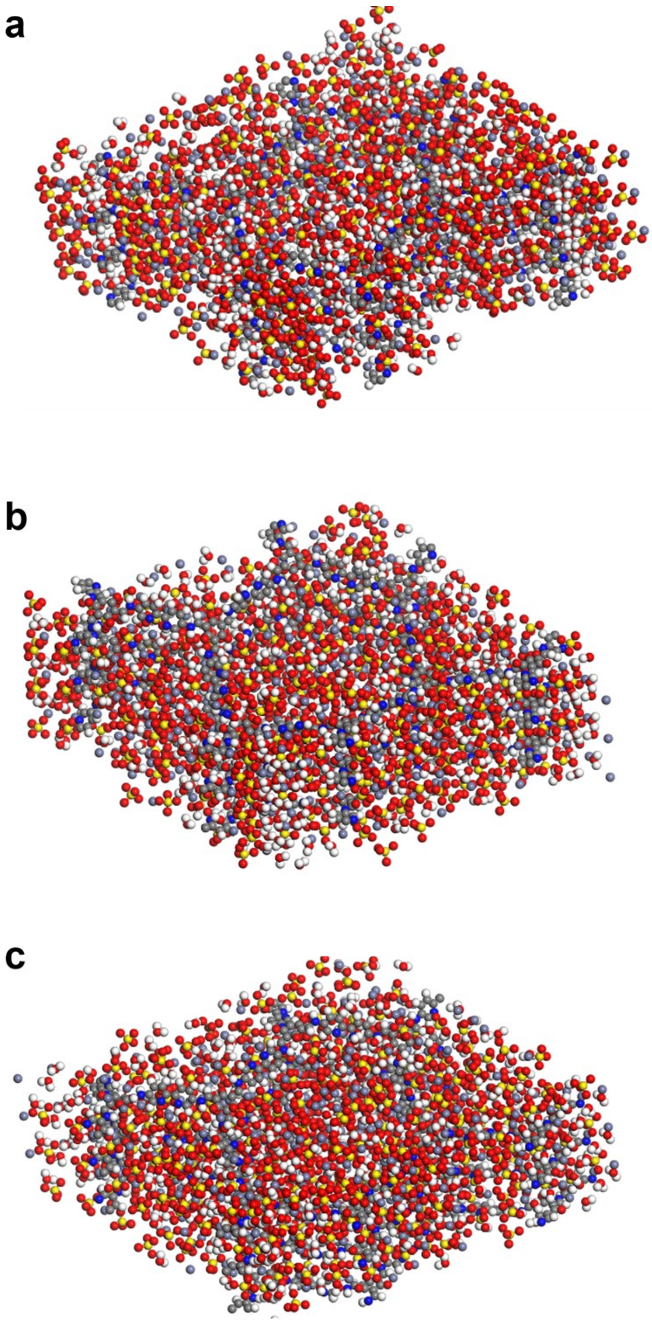

Figure S15. Snapshots from molecular dynamics (MD) simulations of 2 M $ZnSO_4$ with TP- BPY COFs (a) 0 ns, (b) 5 ns, (c) 10 ns.

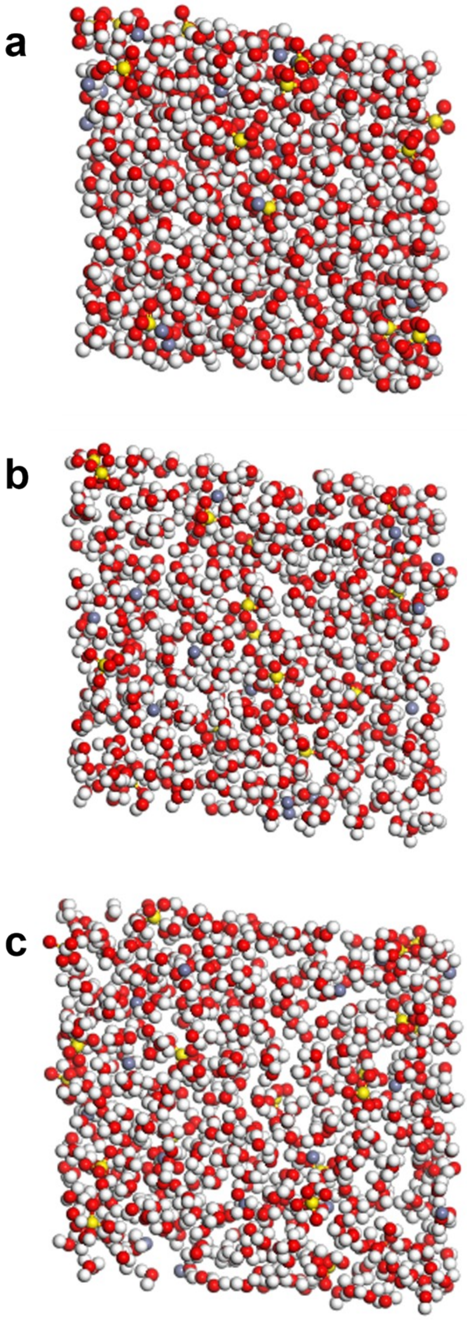

Figure S16. Snapshots from molecular dynamics (MD) simulations of 2 M $ZnSO_4$ (a) 0 ns, (b) 5 ns, (c) 10 ns.

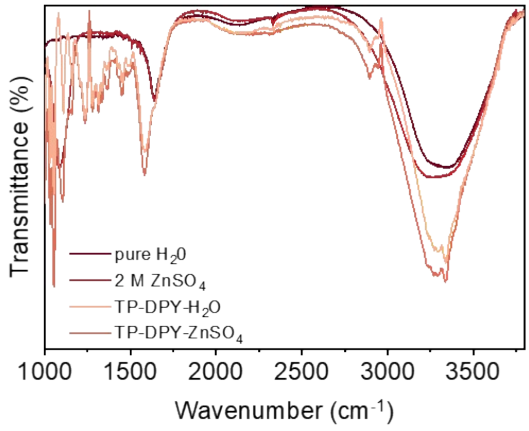

Figure S17. FTIR spectra of $ZnSO_4$ and pure H_2O with TP-DPY membrane.

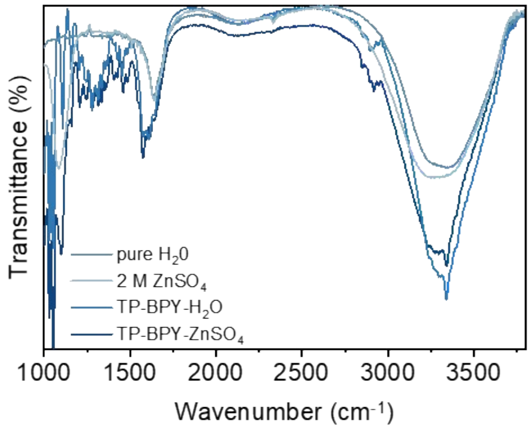

Figure S18. FTIR spectra of $ZnSO_4$ and pure H_2O with TP-BPY membrane.

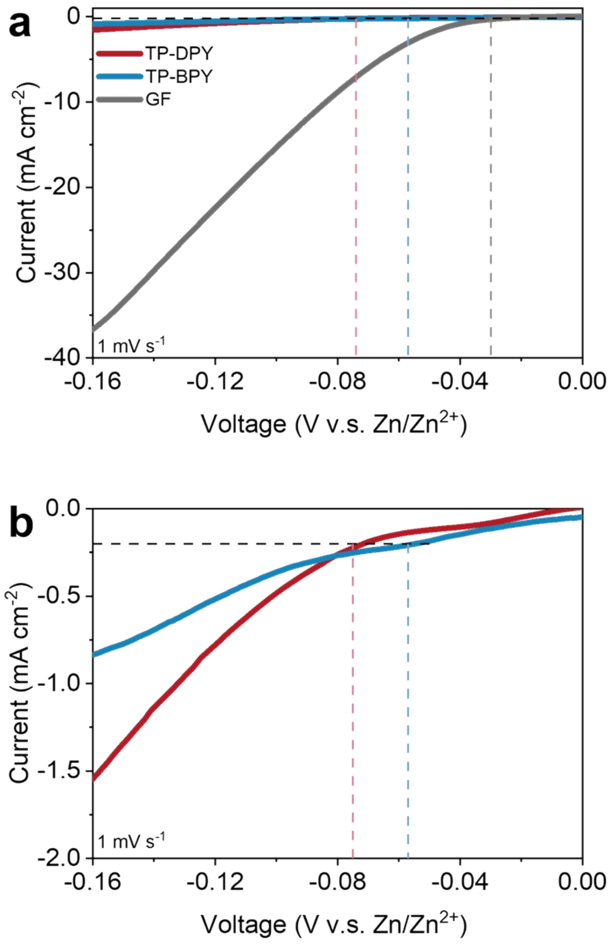

Figure S19. LSV curves of Zn||Zn symmetry cell at 1 mV S^{-1}.

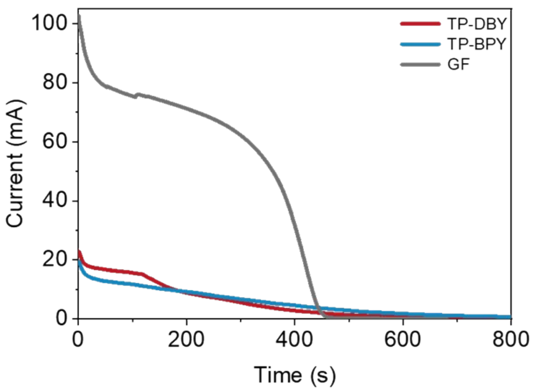

Figure S20. Zn||Cu asymmetric cell CA curves at a bias potential of 150 mV.

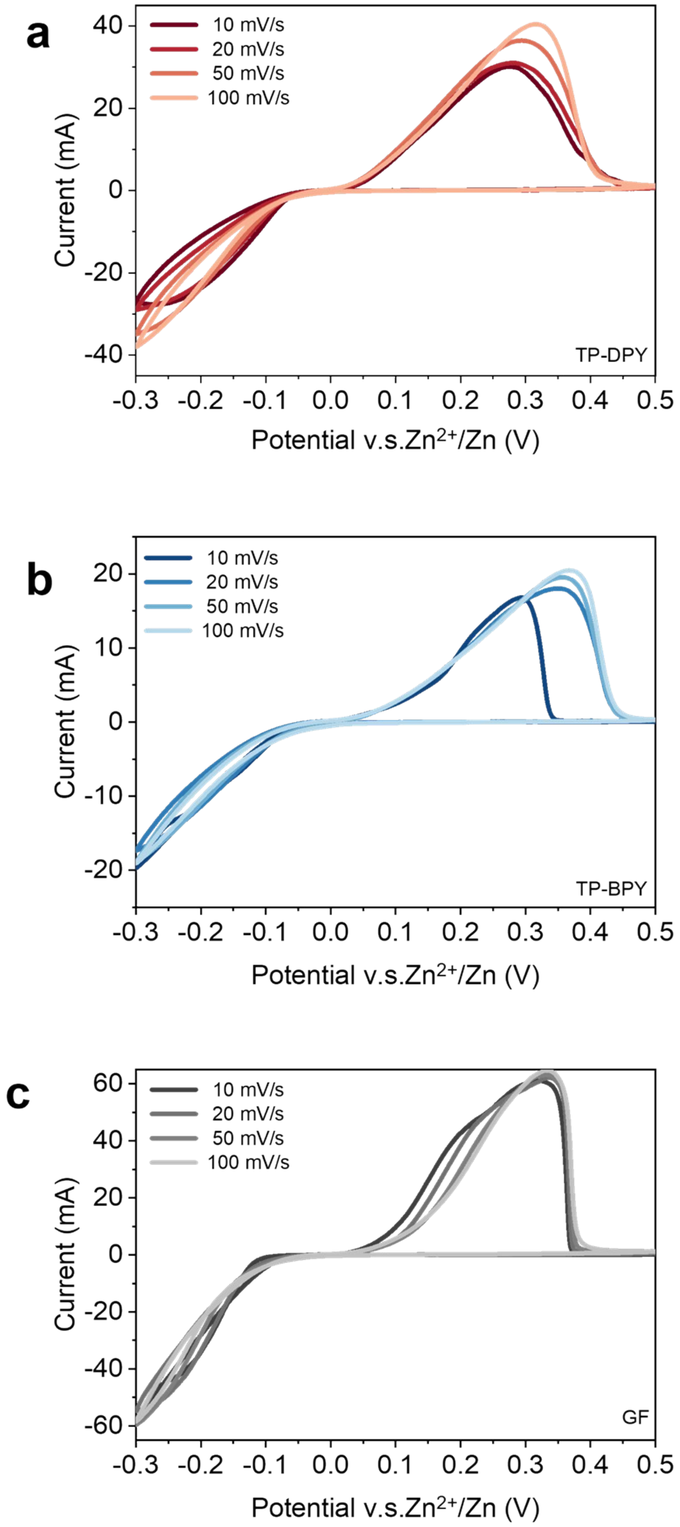

Figure S21. Cyclic voltammetry (CV) curves of Zn||Cu cells with (a) TP-DPY, (b) TP-BPY, and (c) glass fiber (GF) membranes, respectively, at various scanning rates.

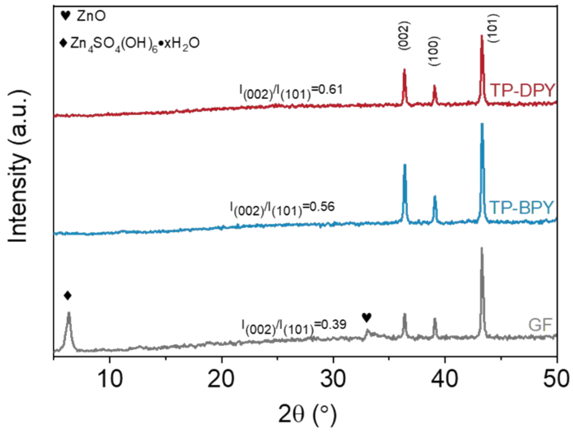

Figure S22. X-ray diffractometer (XRD) patterns of Cu foil after the first deposition cycle of Zn||Cu cells with TP-DPY, TP-BPY, and glass fiber (GF) membranes, respectively (1 mA cm^{-2} and 1 mAh cm^{-2}).

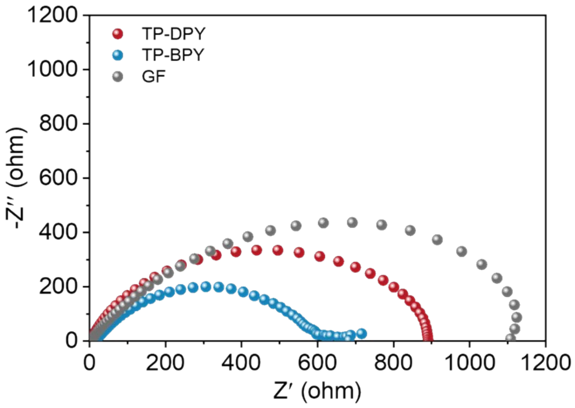

Figure S23. Nyquist plots of Zn||Zn symmetrical cell to calculate W_a.

Table S3. Fitting impendence parameters.

Separator	Rs (Ω)	Rct (Ω)
TP-DPY	2.018	916
TP-BPY	13.6	536.6
GF	4.213	938.3

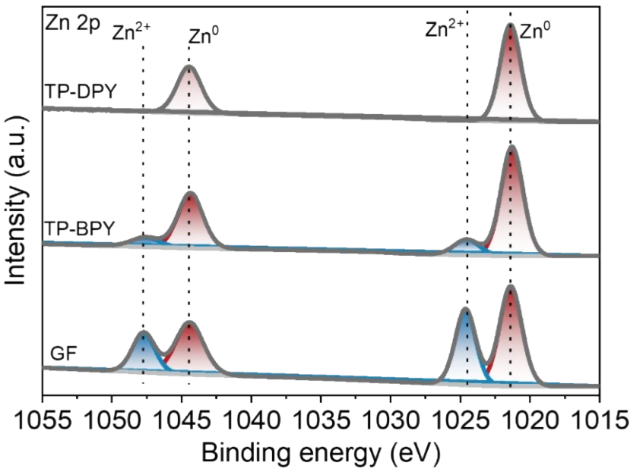

Figure S24. XPS spectra (Zn 2p) of Zn electrode of Zn||Zn symmetric cell assembled by different separators after cycling test.

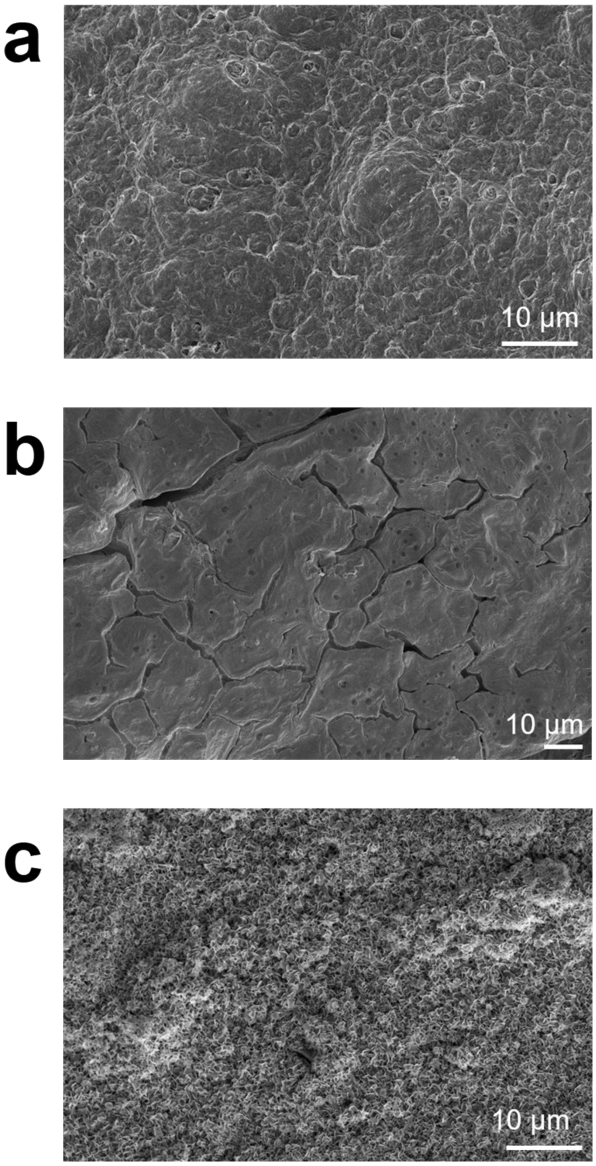

Figure S25. Scanning electron microscopy (SEM) of the deposition Zn images on Cu foil surface after the first deposition cycle of Zn||Cu cells (1 mA cm^{-2} and 1 mAh cm^{-2}) with (a) TP-DPY, (b) TP-BPY, (c) glass fiber (GF).

Table S4. The performance comparison between TD-DPY membrane and other reported separators applied in aqueous zinc battery.

**Materials**	**Zn||Zn Cycle life** (Current density)	**Reference**
COF-Zn	2900 h (0.2 mA cm^{-2})	*Angew. Chem. Int. Ed.* **2025**, 64, e202423118.
NH_2-MIL-125	2000 h (2 mA cm^{-2})	*Adv. Energy Mater.* **2025**, 15, e04201.
Janus COF-OH	1000 h (1 mA cm^{-2})	*Adv. Func. Mater.* **2025**, *e28150.*
UiO-66-GF	1650 h (2 mA cm^{-2})	*Nano-Micro Letters*
TCH	2700 h (1 mA cm^{-2})	*Adv. Func. Mater.* **2024**, *34*, 2405957.
HTS	3000 h (2 mA cm^{-2})	*Nat. Commun.* **2025**, 16, 259.
ZSM-5@GF	2500 h (1 mA cm^{-2})	ACS Nano **2026**, *20*, 4279-4290.
B/CNF	1200 h (1 mA cm^{-2})	*Adv. Energy Mater.* **2025**, *15*, e03368.
SA/HNTs	1800 h (1 mA cm^{-2})	*Adv. Func. Mater.* **2025**, e20280.
FCOF-30	1200 h (10 mA cm^{-2})	*J. Am. Chem. Soc.* **2025,** 147, 36626-36641.
SAS-N	3000 h (1 mA cm^{-2})	*Energy Environ. Sci.* **2025**, 18, 10048-10060.
ZSM-5	2000 h (1 mA cm^{-2})	*Adv. Mater.* **2022**, 34, 2207209
MZS	2400 h (1 mA cm^{-2})	*Adv. Func. Mater.* 2026, 36, e17715.
ZIF	2900 h (0.5 mA cm^{-2})	*Adva. Mater.* **2025**, 37, 2413167.
S-COF@PAN	3000 h (1 mA cm^{-2})	*Adv. Mater.* **2026**, e23580.
TP-DPY	5200 h (1 mA cm^{-2})	This work
TP-DPY	3100 h (5 mA cm^{-2})	This work

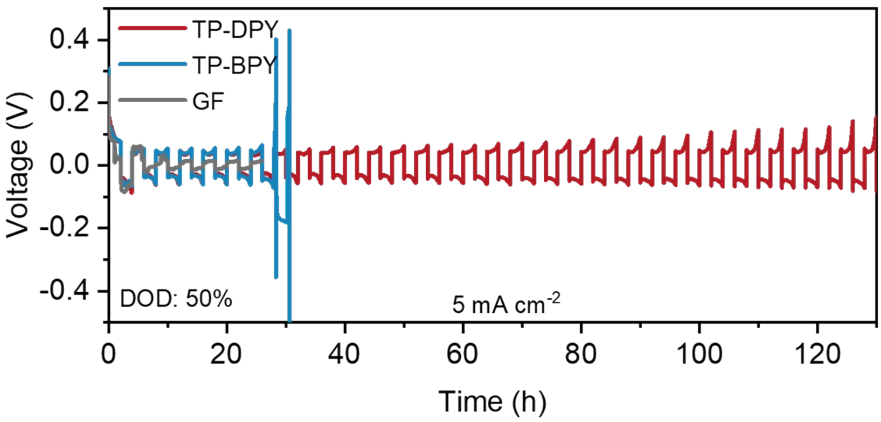

Figure S26. Cycling performance of Zn||Zn symmetry cells with a deep Zn stripping (DOD=50%) under 5 mA cm^{-2}.

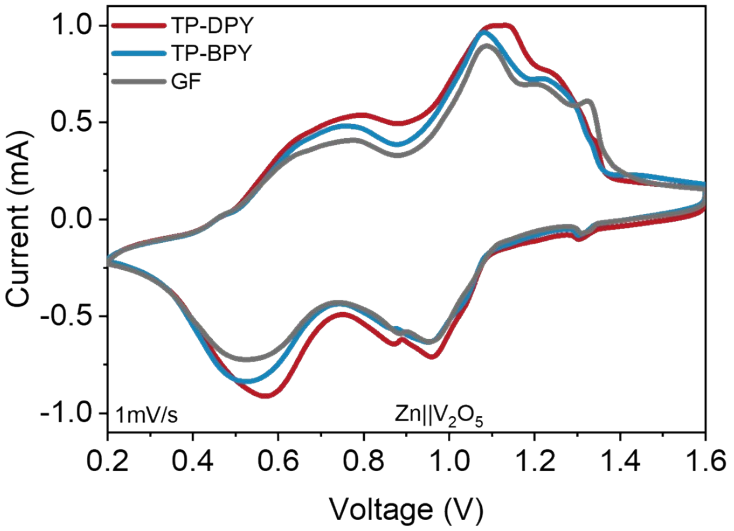

Figure S27. CV curves of Zn||V_2O_5 full cells with different membranes at 1 mV s^{-1}.

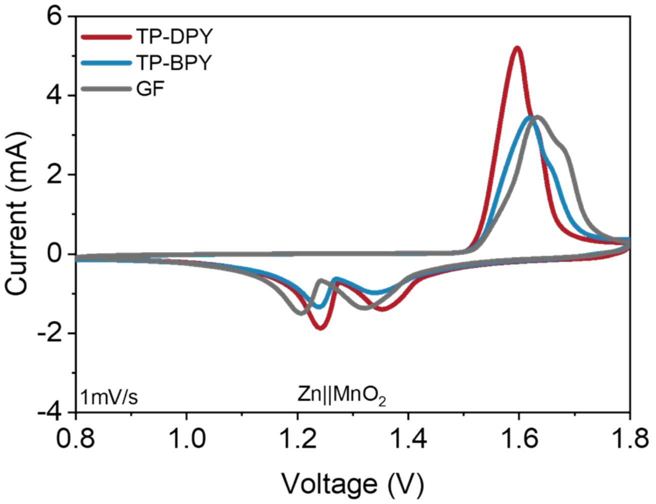

Figure S28. CV curves of Zn||MnO_2 full cells with different membranes at 1 mV s^{-1}.

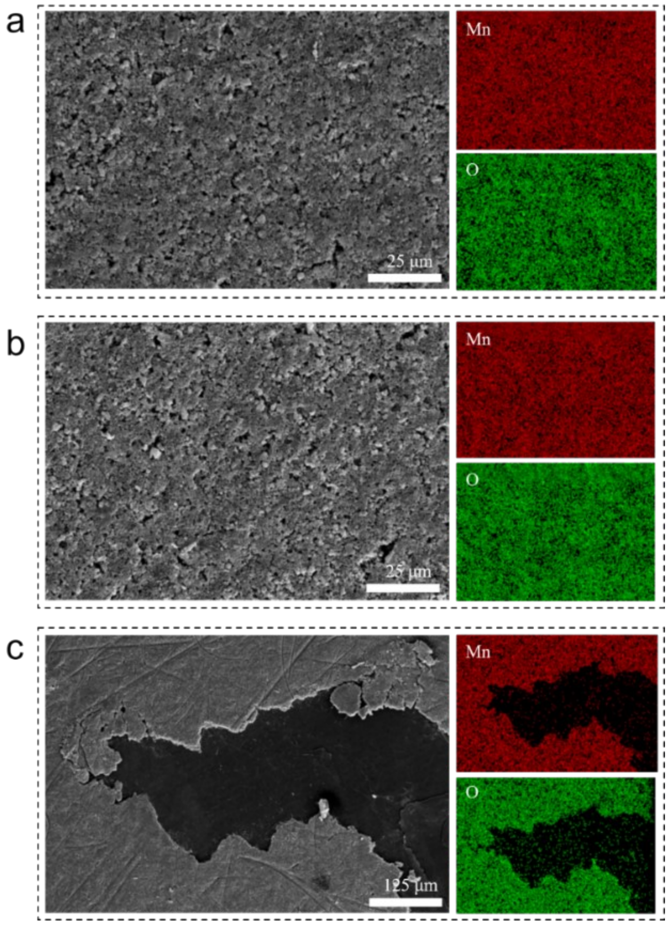

Figure S29. SEM and EDS mapping image of MnO_2 cathode after cycling test in full batteries paired with (a) TP-DPY, (b) TP-BPY and (c) GF separators.

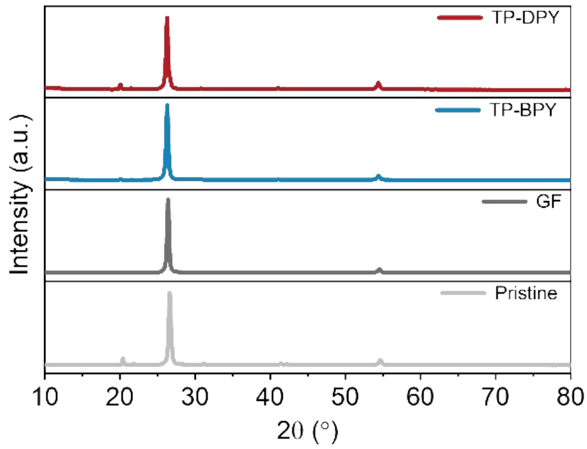

Figure S30. XRD profiles of V_2O_5 cathode after cycling test in full batteries paired with TP-DPY, TP-BPY and GF separators.

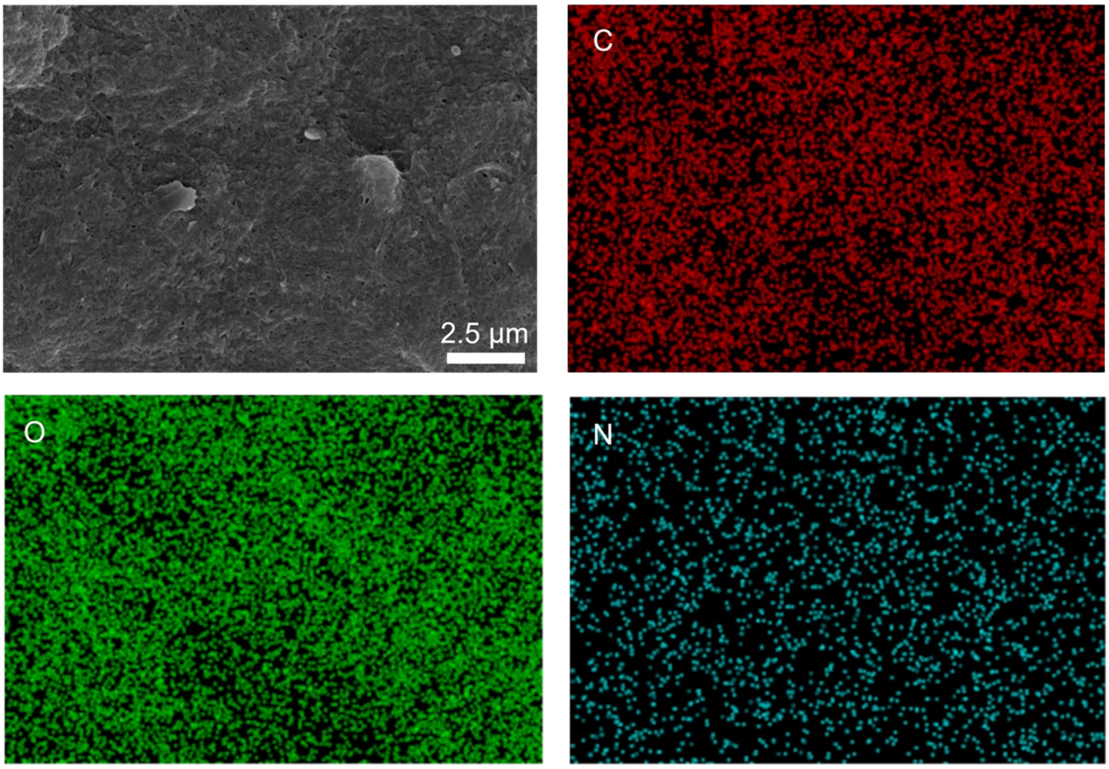

Figure S31. Surface-view scanning electron microscopy (SEM) and corresponding energy X-ray spectroscopy (EDS) mapping images of TP-DPY membrane after 300 cycles at 1 mA cm^{-2}

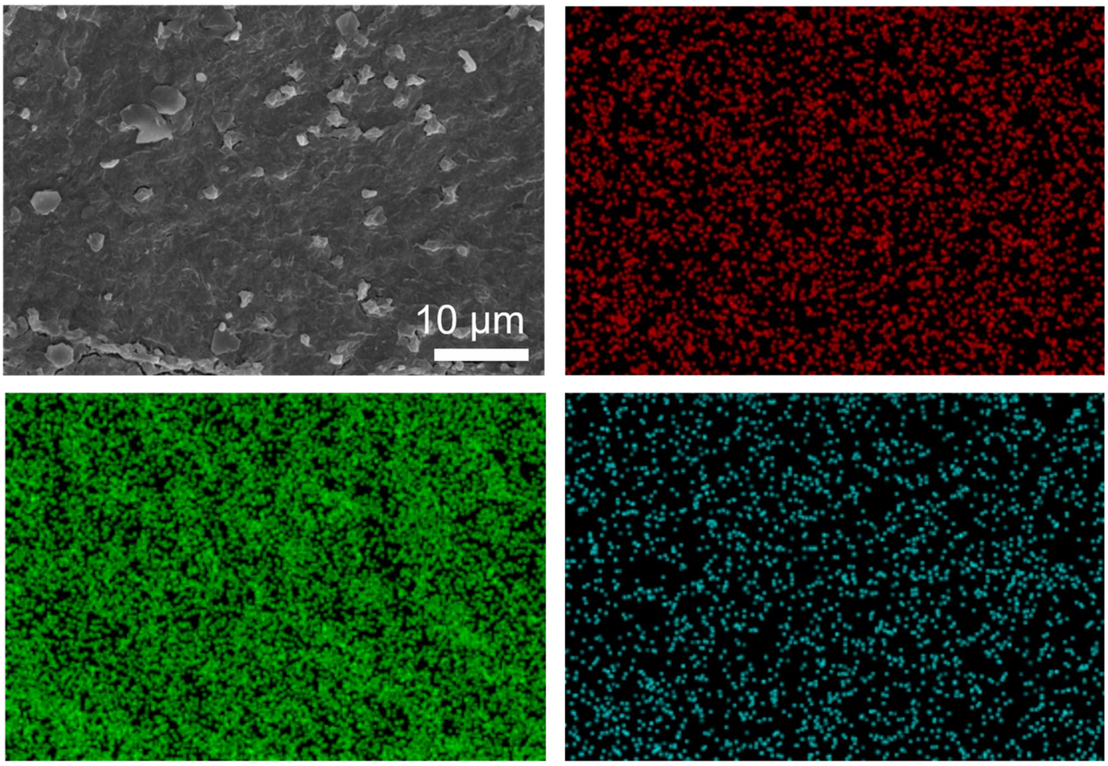

Figure S32. Surface-view scanning electron microscopy (SEM) and corresponding energy X-ray spectroscopy (EDS) mapping images of TP-BPY membrane after 300 cycles at 1 mA cm^{-2}

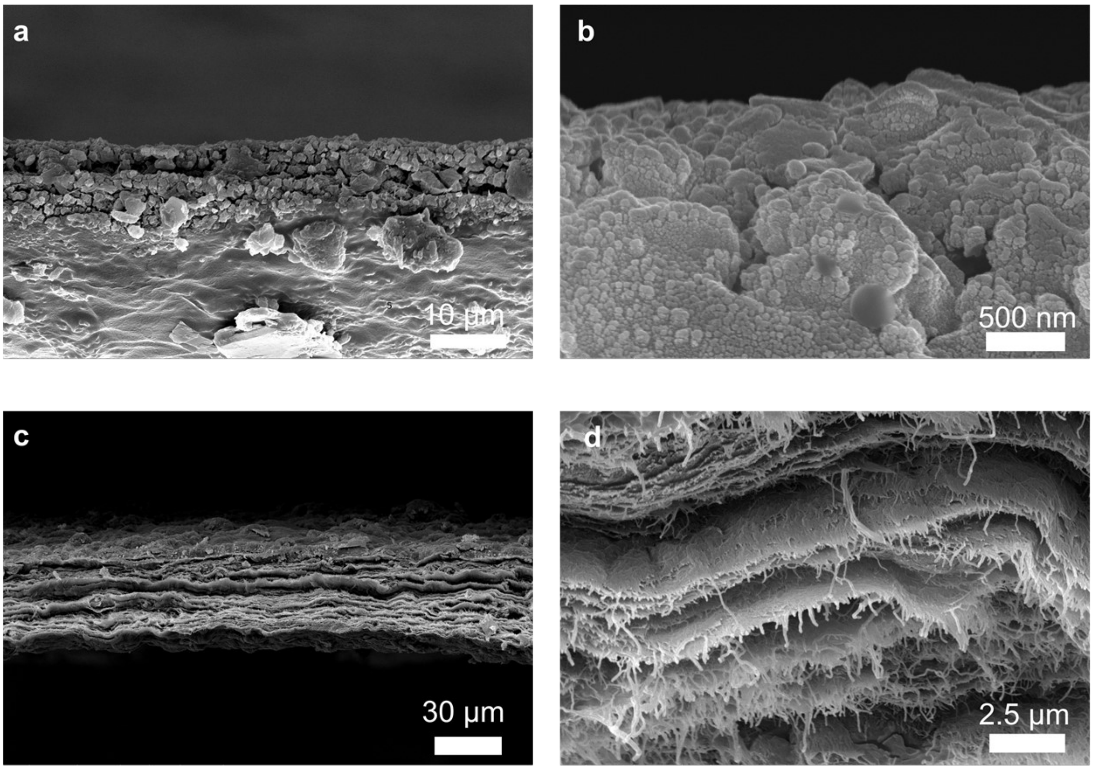

Figure S33. Side-view scanning electron microscopy (SEM) images of (a~b) TP-DPY, (c~d) TP-BPY membrane after 300 cycles at 1 mA cm^{-2}.

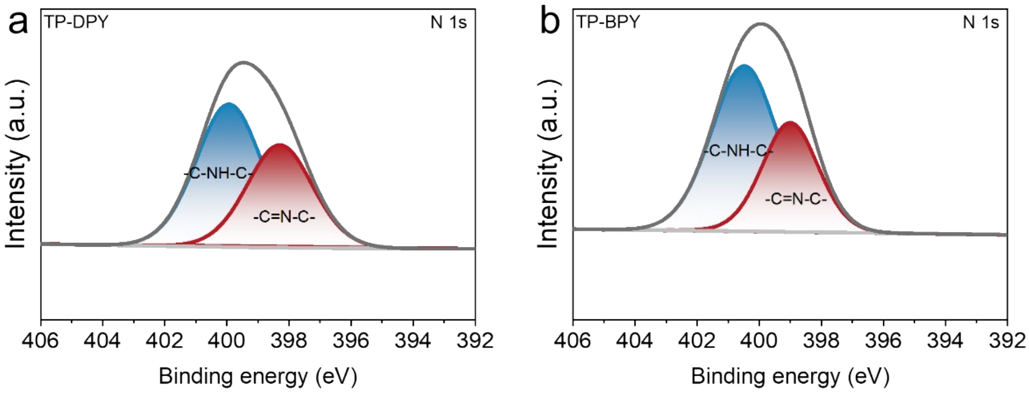

Figure S34. XPS spectra for N 1s of COF membrane (a) TP-DPY, (b) TP-BPY after cycle.

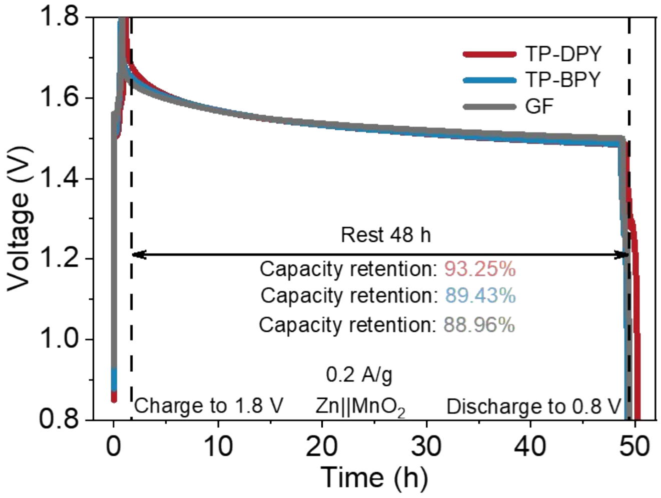

Figure S35. Self-discharge curves of Zn||MnO_2 full cells with different membranes

References

1. Yang, S.; Zhang, Y.; Zhang, Y.; Deng, J.; Chen, N.; Xie, S.; Ma, Y.; Wang, Z., Designing anti-swelling nanocellulose separators with stable and fast ion transport channels for efficient aqueous zinc-ion batteries. *Advanced Functional Materials* **2023,** *33* (42), 2304280.

2. Zheng, Z.; Guo, S.; Yan, M.; Luo, Y.; Cao, F., A functional janus Ag nanowires/bacterial cellulose separator for high-performance dendrite-free zinc anode under harsh conditions. *Advanced Materials* **2023,** *35* (47), 2304667.

3. Leftheriotis, G.; Papaefthimiou, S.; Yianoulis, P., Dependence of the estimated diffusion coefficient of LixWO3 films on the scan rate of cyclic voltammetry experiments. *Solid State Ionics* **2007,** *178* (3-4), 259-263.

4. Jin, S.; Yin, J.; Gao, X.; Sharma, A.; Chen, P.; Hong, S.; Zhao, Q.; Zheng, J.; Deng, Y.; Joo, Y. L., Production of fast-charge Zn-based aqueous batteries via interfacial adsorption of ion-oligomer complexes. *Nature communications* **2022,** *13* (1), 2283.

5. Kim, M.; Lee, J.; Kim, Y.; Park, Y.; Kim, H.; Choi, J. W., Surface overpotential as a key metric for the discharge–charge reversibility of aqueous zinc-ion batteries. *Journal of the American Chemical Society* **2023,** *145* (29), 15776-15787.
